# Scaling limit of the odometer in divisible sandpiles

**DOI:** 10.1007/s00440-017-0821-x

**Published:** 2017-12-08

**Authors:** Alessandra Cipriani, Rajat Subhra Hazra, Wioletta M. Ruszel

**Affiliations:** 1 University of Bath, Bath, UK; 2 Indian Statistical Institute, Kolkata, India; 3 TU Delft, Delft, The Netherlands

**Keywords:** Divisible sandpile, Odometer, Membrane model, Gaussian field, Green’s function, Abstract Wiener space, 31B30, 60J45, 60G15, 82C20

## Abstract

In a recent work Levine et al. (Ann Henri Poincaré 17:1677–1711, 2016. 10.1007/s00023-015-0433-x) prove that the odometer function of a divisible sandpile model on a finite graph can be expressed as a shifted discrete bilaplacian Gaussian field. For the discrete torus, they suggest the possibility that the scaling limit of the odometer may be related to the continuum bilaplacian field. In this work we show that in any dimension the rescaled odometer converges to the continuum bilaplacian field on the unit torus.

## Introduction

The concept of self-organized criticality was introduced in Bak et al. [2] as a lattice model with a fairly elementary dynamics. Despite its simplicity, this model exhibits a very complex structure: the dynamics drives the system towards a stationary state which shares several properties of equilibrium systems at the critical point, e.g. power law decay of cluster sizes and of correlations of the height-variables. The model was generalised by Dhar [5] in the so-called Abelian sandpile model (ASP). Since then, the study of self-criticality has become popular in many fields of natural sciences, and we refer the reader to Járai [10] and Redig [20] for an overview on the subject. In particular, several modifications of the ASP were introduced such as non-Abelian models, ASP on different geometries, and continuum versions like the divisible sandpile treated in Levine and Peres [15, 16]. We are interested in the latter one which is defined as follows. By a graph $G = (V, E)$ we indicate a connected, locally finite and undirected graph with vertex set *V* and edge set *E*. By $\mathrm {deg}(x)$ we denote the number of neighbours of $x\in V$ in *E* and we write “$y\sim _V x$” when $(x,\,y)\in E$. A divisible sandpile configuration on G is a function $s : V \rightarrow {{\mathrm{\mathbb {R}}}}$, where *s*(*x*) indicates a mass of particles at site *x*. Note that here, unlike the ASP, *s*(*x*) is a real-valued (possibly negative) number. If a vertex $x \in V$ satisfies $s(x)> 1$, it topples by keeping mass 1 for itself and distributing the excess $s(x) -1$ uniformly among its neighbours. At each discrete time step, all unstable vertices topple simultaneously.

Given $(\sigma (x))_{x\in V}$ i.i.d. standard Gaussians, we construct the divisible sandpile with weights $(\sigma (x))_{x\in V}$ by defining its initial configuration as

1.1 $$\begin{aligned} s(x)=1+\sigma (x)-\frac{1}{|V|}\sum _{y\in V} \sigma (y). \end{aligned}$$

As in many models of statistical mechanics, one is interested in defining a notion of criticality here too.

Let $e^{(n)}(x)$ denote the total mass distributed by *x* before time *n* to any of its neighbours. If $e^{(n)}(x)\uparrow e_{V}$ where $e_{V}:\, V\rightarrow [0,\,+\infty ]$, then $e_{V}$ is called the *odometer* of *s*. We have the following dichotomy: either $e_{V}<+\infty$ for all $x\in V$ (stabilization), or $e_{V}=+\infty$ for all $x\in V$ (explosion). It was shown in Levine et al. [17] that if *s*(*x*) is assumed to be i.i.d. on an infinite graph which is vertex transitive, and if $\mathsf {E}[s(x)]>1$, *s* does not stabilize, while stabilization occurs for $\mathsf {E}[s(x)]<1$. In the critical case ($\mathsf {E}[s(x)]=1$) the situation is graph-dependent. For an infinite vertex transitive graph, with $\mathsf {E}[s(x)]=1$ and $0<\mathsf {Var}(s(x))<+\infty$ then *s* almost surely does not stabilize.

For a finite connected graph, one can give quantitive estimates and representations for $e_V$. It is shown in Levine et al. [17, Proposition 1.3] that the odometer corresponding to the density (1.1) on a finite graph *V* has distribution

$$\begin{aligned} (e_V(x))_{x\in V}\overset{d}{=}\left( \eta (x) -\min _{z\in V}\eta (z)\right) _{x\in V} \end{aligned}$$

where $\eta$ is a “bilaplacian” centered Gaussian field with covariance

$$\begin{aligned} \mathsf {E}[\eta (x)\eta (y)] =\frac{1}{\mathrm {deg}(x)\mathrm {deg}(y)}\sum _{w\in V}g(x, w)g(w, y) \end{aligned}$$

setting

1.2 $$\begin{aligned} g(x,y)= \frac{1}{|V|}\sum _{z\in V} g^z(x,y) \end{aligned}$$

and $g^z(x, y)=\mathsf {E}\left[ \sum _{m=0}^{\tau _z-1}{{\mathrm{\mathbb {1}}}}_{\{S_m=y\}}\right]$ for $S=(S_m)_{m\ge 0}$ a simple random walk on *V* starting at *x* and $\tau _z := \inf \{m\ge 0 : S_m = z\}$. The field is called “bilaplacian” since a straightforward computation shows that

$$\begin{aligned} \Delta ^2_g\left( \frac{1}{\mathrm {deg}(x)\mathrm {deg}(y)}\sum _{w\in V}g(x, w)g(w, y)\right) =\delta _x(y)-\frac{1}{|V|} \end{aligned}$$

where $\Delta _g$ denotes the graph Laplacian

$$\begin{aligned} \Delta _g f(x):=\sum _{y\sim _V x}f(y)-f(x),\quad f:\,V\rightarrow {{\mathrm{\mathbb {R}}}}. \end{aligned}$$

Hence the covariance is related to the Green’s function of the discrete bilaplacian (or biharmonic) operator.

The interplay between the odometer of the sandpile and the bilaplacian becomes more evident in the observation made by Levine et al. on the odometer in $V:={{\mathrm{\mathbb {Z}}}}_n^d$, the discrete torus of side length $n>0$ in dimension *d*. They write (after the statement of Proposition 1.3):

“We believe that if $\sigma$ is identically distributed with zero mean and finite variance, then the odometer, after a suitable shift and rescaling, converges weakly as $n\rightarrow + \infty$ to the bilaplacian Gaussian field on ${{\mathrm{\mathbb {R}}}}^d$”.

Note that, although they work with Gaussian weights in the proof of Proposition 1.3, their comment comprises also the case when $\sigma$ has a more general distribution. Inspired by the above remark, we determine the scaling limit of the odometer in $d\ge 1$ for general i.i.d. weights: we show that indeed it equals $\Xi$, the continuum bilaplacian, but on the unit torus $\mathbb {T}^d$ (see Theorems 1 and 2). A heuristic for the toric limit is that the laplacian we consider is on ${{\mathrm{\mathbb {Z}}}}_n^d$, which can be seen as dilation of the discrete torus $\mathbb {T}^d\cap (n^{-1}{{\mathrm{\mathbb {Z}}}})^d$. We highlight that $\Xi$ is not a random variable, but a *random distribution* living in an appropriate Sobolev space on $\mathbb {T}^d$. There are several ways in which one can represent such a field: a convenient one is to let $\Xi$ be a collection of centered Gaussian random variables $\left\{ \left\langle \Xi ,\,u \right\rangle :\,u\in H^{-1}(\mathbb {T}^d)\right\}$ with variance $\mathsf {E}\left[ \left\langle \Xi ,\,u \right\rangle ^2\right] =\Vert u\Vert ^2_{{-1}}$, where

$$\begin{aligned} \Vert u\Vert ^2_{{-1}}:=\left( u,\Delta ^{-2}u\right) _{L^2(\mathbb {T}^d)} \end{aligned}$$

and $\Delta ^2$ now is the continuum bilaplacian operator. We will give the analytical background to this definition in Sect. 2.2. As a by-product of our proof, we are able to determine the kernel of the continuum bilaplacian on the torus which, to the best of the authors’ knowledge, is not explicitly stated in the literature.

*Related work* Scaling limits for sandpiles have already been investigated: in the ASP literature limits for stable configurations have been studied, for example, in Levine et al. [18] and Pegden and Smart [19]. Their works are concerned with the partial differential equation that characterizes the scaling limit of the ASP in ${{\mathrm{\mathbb {Z}}}}^2$. They also provide an interesting explanation of the fractal structure which arises when a large number of chips are placed at the origin and allowed to topple. The properties of the odometer play an important role in their analysis. In the literature of divisible sandpiles models, the scaling limit of the odometer was determined for an $\alpha$-stable divisible sandpile in Frómeta and Jara [6], who deal with a divisible sandpile for which mass is distributed not only to nearest-neighbor sites, but also to “far away” ones. Their limit is related to an obstacle problem for the truncated fractional Laplacian. In the subsequent work Cipriani et al. [4], the authors of the present paper extend the result to the case in which the assumption on the finite variance of the $\sigma$’s is relaxed, and obtain an alpha-stable generalised field in the scaling limit.

The discrete bilaplacian (also called *membrane*) model was introduced in Sakagawa [23] and Kurt [11, 12] for the box of ${{\mathrm{\mathbb {Z}}}}^d$ with zero boundary conditions. In $d\ge 4$ Sun and Wu [27] and Lawler et al. [13] construct a discrete model for the bilaplacian field by assigning random signs to each component of the uniform spanning forest of a graph and study its scaling limit. As far as the authors know, Levine et al. [17] is the first paper in which the discrete bilaplacian model has been considered with periodic boundary conditions.

### Main results

*Notation* We start with some preliminary notations which are needed throughout the paper. Let $\mathbb {T}^d$ be the *d*-dimensional torus, alternatively viewed as $\frac{{{\mathrm{\mathbb {R}}}}^d}{{{\mathrm{\mathbb {Z}}}}^d}$ or as $[-\frac{1}{2},\,\frac{1}{2})^d\subset {{\mathrm{\mathbb {R}}}}^d$. ${{\mathrm{\mathbb {Z}}}}_n^d:=[-\frac{n}{2},\,\frac{n}{2}]^d\cap {{\mathrm{\mathbb {Z}}}}^d$ is the discrete torus of side-length $n\in {{\mathrm{\mathbb {N}}}}$, and $\mathbb {T}_n^d:=[-\frac{1}{2},\,\frac{1}{2}]^d\cap (n^{-1}{{\mathrm{\mathbb {Z}}}})^d$ is the discretization of $\mathbb {T}^d$. Moreover let $B(z,\,\rho )$ a ball centered at *z* of radius $\rho >0$ in the $\ell ^\infty$-metric. We will use throughout the notation $z\cdot w$ for the Euclidean scalar product between $z,\,w\in {{\mathrm{\mathbb {R}}}}^d$. With $\Vert \,\cdot \,\Vert _\infty$ we mean the $\ell ^\infty$-norm, and with $\Vert \cdot \Vert$ the Euclidean norm. We will let $C,\,c$ be positive constants which may change from line to line within the same equation. We define the Fourier transform of a function $u\in L^1(\mathbb {T}^d)$ as ${\widehat{u}}(y):=\int _{\mathbb {T}^d}u(z)\exp \left( -2\pi \iota y\cdot z\right) {{\mathrm{d}}}z$ for $y\in {{\mathrm{\mathbb {Z}}}}^d$. We will use the symbol $\,\widehat{\cdot }\,$ to denote also Fourier transforms on ${{\mathrm{\mathbb {Z}}}}_n^d$ and ${{\mathrm{\mathbb {R}}}}^d$. We will say that a function $f(n)=\mathrm {o}\left( 1\right)$ if $\lim _{n\rightarrow +\infty }f(n)=0$.

We can now state our main theorem: we consider the piecewise interpolation of the odometer on small boxes of radius $\frac{1}{2n}$ and show convergence to the continuum bilaplacian field.

#### Theorem 1

(Scaling limit of the odometer for Gaussian weights) Let $d\ge 1$ and let $(\sigma (x))_{x\in {{\mathrm{\mathbb {Z}}}}_n^d}$ be a collection of i.i.d. standard Gaussians. Let $e_n(\cdot ):=e_{{{\mathrm{\mathbb {Z}}}}_n^d}(\cdot )$ be the odometer on ${{\mathrm{\mathbb {Z}}}}_n^d$ associated to these weights. The formal field

1.3 $$\begin{aligned} \Xi _n(x):=4\pi ^2\sum _{z\in \mathbb {T}^d_n}n^{\frac{d-4}{2}}e_n({nz}){{\mathrm{\mathbb {1}}}}_{B\left( z,\,\frac{1}{2n}\right) }(x),\quad x\in \mathbb {T}^d \end{aligned}$$

converges in law as $n\rightarrow +\infty$ to the bilaplacian field $\Xi$ on $\mathbb {T}^d$. The convergence holds in the Sobolev space ${\mathcal {H}}_{-\epsilon }(\mathbb {T}^d)$ with the topology induced by the norm $\Vert \cdot \Vert _{{{\mathcal {H}}}_{-\epsilon }(\mathbb {T}^d)}$ for any $\epsilon >\max \left\{ 1+\frac{d}{4},\,\frac{d}{2}\right\}$ (see Sect. 2.2 for the analytic specifications).

The reason to impose $\epsilon >\max \left\{ 1+\frac{d}{4},\,\frac{d}{2}\right\}$ is two-folded: on the one hand, it ensures the tightness of $\Xi _n$, on the other it allows us to define the law of $\Xi$ properly (see the construction of abstract Wiener space in Sect. 2.2). Observe moreover that $\max \left\{ 1+\frac{d}{4},\,\frac{d}{2}\right\}$ has a transition at $d=4$, which is reminiscent of the phase transition of the bilaplacian model on ${{\mathrm{\mathbb {Z}}}}^d$ (see for instance Kurt [12]).

We can now show the next Theorem, which generalises the previous one to the case in which the weights have an arbitrary distribution with mean zero and finite variance. We keep the proof separate from the Gaussian one, as the latter will allow us to obtain precise results on the kernel of the bilaplacian, and has also a different flavor. Moreover, the more general proof relies on estimates we obtain in the Gaussian case. With a slight abuse of notation, we will define a field $\Xi _{n}$ as in Theorem 1 also for weights which are not necessarily Gaussian (in the sequel, it will be clear from the context to which weights we are referring to).

#### Theorem 2

(Scaling limit of the odometer for general weights) Assume $(\sigma (x))_{x\in {{\mathrm{\mathbb {Z}}}}_n^d}$ is a collection of i.i.d. variables with $\mathsf {E}\left[ \sigma \right] =0$ and $\mathsf {E}\left[ \sigma ^2\right] =1$. Let $d\ge 1$ and $e_n(\cdot )$ be the corresponding odometer. If we define the formal field $\Xi _n$ as in (1.3) for such weights, then it converges in law as $n\rightarrow +\infty$ to the bilaplacian field $\Xi$ on $\mathbb {T}^d$. The convergence holds in the same fashion of Theorem 1.

We now give an explicit description of the covariance structure of $\Xi$. Our motivation is also a comparison with the whole-space bilaplacian field already treated in the literature. More precisely, for $d\ge 5$, Sun and Wu [27, Definition 3] define the bilaplacian field ${\widetilde{\Xi }}_d$ on ${{\mathrm{\mathbb {R}}}}^d$ as the unique distribution on $\left( C^\infty _c({{\mathrm{\mathbb {R}}}}^d)\right) ^*$ such that, for all $u\in C^\infty _c({{\mathrm{\mathbb {R}}}}^d)$, $\left\langle {\widetilde{\Xi }}_d,\,u \right\rangle$ is a centered Gaussian variable with variance

$$\begin{aligned} \mathsf {E}\left[ \left\langle {\widetilde{\Xi }}_d,\,u \right\rangle ^2\right] =\iint _{{{\mathrm{\mathbb {R}}}}^d\times {{\mathrm{\mathbb {R}}}}^d}u(x)u(y)\Vert x-y\Vert ^{4-d}{{\mathrm{d}}}x {{\mathrm{d}}}y. \end{aligned}$$

Since we obtain a limiting field on $\mathbb {T}^d$, we think it is interesting to give a representation for the covariance kernel of the biharmonic operator in our setting. From now on, when we use the terminology “zero average” for a function *u*, we always mean $\int _{\mathbb {T}^d}u(x){{\mathrm{d}}} x=0.$

#### Theorem 3

(Kernel of the biharmonic operator in higher dimensions) Let $d\ge 5$. Let furthermore $u \in C^\infty (\mathbb {T}^d)$ and with zero average. Then there exists ${\mathcal {G}}_d\in L^1({{\mathrm{\mathbb {R}}}}^d)$ such that

1.4 $$\begin{aligned} \mathsf {E}\left[ \left\langle \Xi _,\,u \right\rangle ^2\right]&=\left( u,\,\Delta ^{-2}u\right) _{L^2(\mathbb {T}^d)}\nonumber \\&=\iint _{\mathbb {T}^d\times \mathbb {T}^d} u(z)u(z')\sum _{w\in {{\mathrm{\mathbb {Z}}}}^d}{\mathcal {G}}_d(z-z'+w){{\mathrm{d}}}z{{\mathrm{d}}}z'. \end{aligned}$$

${\mathcal {G}}_d$ can be computed as follows: there exists $h_d\in C^\infty ({{\mathrm{\mathbb {R}}}}^d)$ depending on *d* such that

1.5 $$\begin{aligned} {\mathcal {G}}_d(\,\cdot \,)= {\pi ^{4-\frac{d}{2}}\Gamma \left( \frac{d-4}{2}\right) } \Vert \cdot \Vert ^{4-d}+h_d(\,\cdot \,). \end{aligned}$$

#### Remark 1

(Kernel of the biharmonic operator in lower dimensions) The convergence result of Theorem 2 allows us to determine the kernel in $d\le 3$ too. In fact, for such *d* interchanging sum and integrals is possible, so that we can write

1.6 $$\begin{aligned} \left( u,\,\Delta ^{-2}u\right) _{L^2(\mathbb {T}^d)}=\sum _{\nu \in {{\mathrm{\mathbb {Z}}}}^d{\setminus }\{0\}}\frac{\left| {\widehat{u}}(\nu )\right| ^2}{\Vert \nu \Vert ^{4}}=\iint _{\mathbb {T}^d\times \mathbb {T}^d}u(z)u(z'){\mathcal {K}}(z-z'){{\mathrm{d}}}z{{\mathrm{d}}}z', \end{aligned}$$

where we can define the kernel of the bilaplacian to be

$$\begin{aligned} {\mathcal {K}}(z-z'):=\sum _{\nu \in {{\mathrm{\mathbb {Z}}}}^d{\setminus }\{0\}}\frac{{{\mathrm{e}}}^{2\pi \iota (z-z')\cdot \nu }}{\Vert \nu \Vert ^{4}},\quad z,\,z'\in \mathbb {T}^d. \end{aligned}$$

*Outline of the article*The necessary theoretical background is given in Sect. 2, together with an outline of the strategy of the proof of Theorem 1. Auxiliary results and estimates are provided in Sect. 3. The proof of Theorem 1 lies in Sect. 4, and of Theorem 2 in Sect. 5. Finally we conclude with the proof of Theorem  3 in Sect. 6.

## Preliminaries

In this section we review the basics of the spectral theory of the Laplacian on the discrete torus from Levine et al. [17]. We also remind the fundamentals of abstract Wiener spaces which enable us to construct standard Gaussian random variables on a Sobolev space on $\mathbb {T}^d$. The presentation is inspired by Silvestri [25]. We also comment on the basic strategy of the proof of Theorem 1 and make some important remarks on the test functions we use for our calculations. We refer for the Fourier analytic details used in this article to Stein and Weiss [26] and for a survey on random distributions to Gel’fand and Vilenkin [7].

### Fourier analysis on the torus

We now recall a few facts about the eigenvalues of the Laplacian from Levine et al. [17] for completeness. Consider the Hilbert space $L^2({{\mathrm{\mathbb {Z}}}}_n^d)$ of complex valued functions on the discrete torus endowed with the inner product

$$\begin{aligned} \left\langle f, g \right\rangle = \frac{1}{n^d} \sum _{x\in {{\mathrm{\mathbb {Z}}}}_n^d} f(x)\overline{g(x)}. \end{aligned}$$

The Pontryagin dual group of ${{\mathrm{\mathbb {Z}}}}_n^d$ is identified again with ${{\mathrm{\mathbb {Z}}}}_n^d$. Let $\{ \psi _a: a\in {{\mathrm{\mathbb {Z}}}}_n^d\}$ denote the characters of the group where $\psi _a(x)= \exp (2\pi \iota x\cdot \frac{a}{n})$. The eigenvalues of the Laplacian $\Delta _g$ on discrete tori are given by

$$\begin{aligned} \lambda _w= -4 \sum _{i=1}^d \sin ^2\left( \frac{\pi w_i}{n}\right) ,\quad w\in {{\mathrm{\mathbb {Z}}}}_n^d. \end{aligned}$$

Recalling (1.2), we use the shortcut $g_x(y):= g(y,x)$. Let ${\widehat{g}}_x$ denote the Fourier transform of $g_x$. It follows that

2.1 $$\begin{aligned} {\widehat{g}}_x(0)= n^{-d}\sum _{y\in {{\mathrm{\mathbb {Z}}}}_n^d} g_x(y) =:L \end{aligned}$$

for all $x\in {{\mathrm{\mathbb {Z}}}}_n^d$ (it can be seen in several ways, for example by translation invariance, that *L* is independent of *x*). Finally, we recall Levine et al. [17, Equation (20)]: for all $a\ne 0$,

2.2 $$\begin{aligned} \lambda _a \widehat{g_x}(a)=-2d n^{-d}\psi _{-a}(x). \end{aligned}$$

### Gaussian variables on homogeneous Sobolev spaces on the torus

Since our conjectured scaling limit is a random distribution, we think it is important to keep the article self-contained and give a brief overview of analytic definitions needed to construct the limit in an appropriate functional space. Our presentation is based on Sheffield [24, Section 2] and Silvestri [25, Sections 6.1, 6.2].

An *abstract Wiener space* (AWS) is a triple $(H, B, \mu )$, where:

1. $(H, (\cdot ,\cdot )_H)$ is a Hilbert space,
2. $(B, \Vert \cdot \Vert _{B})$ is the Banach space completion of *H* with respect to the measurable norm $\Vert \cdot \Vert _B$ on *H*, equipped with the Borel $\sigma$-algebra ${\mathcal {B}}$ induced by $\Vert \cdot \Vert _B$, and
3. $\mu$ is the unique Borel probability measure on $(B,{\mathcal {B}})$ such that, if $B^*$ denotes the dual space of *B*, then $\mu \circ \phi ^{-1}\sim {\mathcal {N}}(0, \Vert {\widetilde{\phi }}\Vert ^2_{H})$ for all $\phi \in B^*$, where ${\widetilde{\phi }}$ is the unique element of *H* such that $\phi (h)=({\widetilde{\phi }}, h)_H$ for all $h\in H$.

We remark that, in order to construct a measurable norm $\Vert \cdot \Vert _B$ on *H*, it suffices to find a Hilbert–Schmidt operator *T* on *H*, and set $\Vert \cdot \Vert _B :=\Vert T\cdot \Vert _H$.

Let us construct then an appropriate AWS. Choose $a\in {{\mathrm{\mathbb {R}}}}$. Let us define the operator $(-\Delta )^a$ acting on $L^2(\mathbb {T}^d)$-functions *u* with Fourier series $\sum _{\nu \in {{\mathrm{\mathbb {Z}}}}^d}{\widehat{u}}(\nu ){\mathbf {e}}_{\nu }(\cdot )$ as follows ($({\mathbf {e}}_\nu )_{\nu \in {{\mathrm{\mathbb {Z}}}}^d}$ denotes the Fourier basis of $L^2(\mathbb {T}^d)$):

$$\begin{aligned} (-\Delta )^a \left( \sum _{\nu \in {{\mathrm{\mathbb {Z}}}}^d}{\widehat{u}}(\nu ){\mathbf {e}}_{\nu }\right) (\vartheta )=\sum _{\nu \in {{\mathrm{\mathbb {Z}}}}^d{\setminus }\{0\}}\Vert \nu \Vert ^{2a}{\widehat{u}}(\nu ){\mathbf {e}}_{\nu }(\vartheta ). \end{aligned}$$

Let “$\sim$” be the equivalence relation on $C^\infty (\mathbb {T}^d)$ which identifies two functions differing by a constant and let $H^a(\mathbb {T}^d)$ be the Hilbert space completion of $C^\infty (\mathbb {T}^d)/{\sim }$ under the norm

$$\begin{aligned} (f,\,g)_{a}:=\sum _{\nu \in {{\mathrm{\mathbb {Z}}}}^d{\setminus }\{0\}}\Vert \nu \Vert ^{4a}\widehat{f}(\nu ){\widehat{g}}(\nu ). \end{aligned}$$

Define the Hilbert space

$$\begin{aligned} {\mathcal {H}}_{a}:=\left\{ u\in L^2(\mathbb {T}^d):\,(-\Delta )^a u\in L^2(\mathbb {T}^d)\right\} /{\sim }. \end{aligned}$$

We equip ${\mathcal {H}}_{a}$ with the norm

$$\begin{aligned} \Vert u\Vert _{{\mathcal {H}}_{a}(\mathbb {T}^d)}^2=\left( (-\Delta )^a u,\,(-\Delta )^a u\right) _{L^2(\mathbb {T}^d)}. \end{aligned}$$

In fact, $(-\Delta )^{-a}$ provides a Hilbert space isomorphism between ${{\mathcal {H}}}_{a}$ and $H^a(\mathbb {T}^d)$, which when needed we identify. For

2.3 $$\begin{aligned} b<a-\frac{d}{4} \end{aligned}$$

one shows that $(-\Delta )^{b-a}$ is a Hilbert–Schmidt operator on $H^a$ (cf. also Silvestri [25, Proposition 5]). In our case, we will be setting $a:=-1$. Therefore, by (2.3), for any $-\epsilon :=b<0$ which satisfies $\epsilon >1+\frac{d}{4}$, we have that $(H^{-1},\,\mathcal {H}_{-\epsilon },\,\mu _{-\epsilon })$ is an AWS. The measure $\mu _{-\epsilon }$ is the unique Gaussian law on ${{\mathcal {H}}}_{-\epsilon }$ whose characteristic functional is

$$\begin{aligned} \Phi (u):=\exp \left( -\frac{\Vert u\Vert _{{-1}}^2}{2}\right) . \end{aligned}$$

The field associated to $\Phi$ will be called $\Xi$ and is the limiting field claimed in Theorem 1.

There is a perhaps more explicit description of $\Xi$ which is based on *Gaussian Hilbert spaces* [9, Chapter 1]. The construction is taken from Janson [9, Example 1.25]. Let $(\Omega ,\,{\mathcal A},\,P)$ be a probability space with $\mathcal A$ its Borel $\sigma$-algebra. Assume that on $\Omega$ one can define a sequence of i.i.d. standard Gaussians $({X}_m)_{m\in {{\mathrm{\mathbb {N}}}}}$. Let further $(\mathbf {X}_m)_{m\in {{\mathrm{\mathbb {N}}}}}$ be an orthonormal basis of $H^{-1}(\mathbb {T}^d)$. Then there is an isometric embedding $\Xi :\,H^{-1}(\mathbb {T}^d)\hookrightarrow L^2(\Omega ,\,P)$ such that $\left\langle \Xi ,\,\mathbf X_m\right\rangle {\mathop {=}\limits ^{d}}X_m$ for all *m*. Indeed, by the properties of AWS, the mapping $(H^{-\epsilon })^*\ni \phi \mapsto \left\langle \Xi ,\,\phi \right\rangle$ is an isometry of the dense subspace $(H^{-\epsilon })^*$ onto $S:=\left\{ \left\langle \Xi ,\,u\right\rangle :\,u\in (H^{-\epsilon })^*\right\}$. The mapping can be extended by continuity to an isometry between $H^{-1}$ and the corresponding closure of *S*. Taking $\Omega :={{\mathcal {H}}}_{-\epsilon }$ and $P:=\mu _{-\epsilon }$, this entails an alternative construction of $\Xi$: it is the unique Gaussian process indexed by $H^{-1}$ such that $\Xi {\mathop {=}\limits ^{d}}\left\{ \left\langle \Xi ,\,u\right\rangle :\,u\in H^{-1}(\mathbb {T}^d)\right\}$ with $\left\langle \Xi ,\,u\right\rangle \sim {\mathcal {N}}\left( 0,\,\Vert u\Vert _{-1}^2\right)$ for any $u\in H^{-1}(\mathbb {T}^d).$

### Strategy of the proof of Theorem 1

Firstly, we show that $\eta$ can be decomposed into the sum of two independent fields, namely

#### Proposition 4

There exist a centered Gaussian field $(\chi _x)_{x\in {{\mathrm{\mathbb {Z}}}}_n^d}$ with covariance $\mathsf {E}[\chi _x\chi _y]=H(x,y)$ as in (3.3) and a centered normal random variable *Y* with variance $(2d)^{-2} n^d L^2$ (where *L* is as in (2.1)), such that *Y* is independent from $(\chi _x)_{x\in {{\mathrm{\mathbb {Z}}}}_n^d}$ and

$$\begin{aligned} (\eta (x))_{x\in {{\mathrm{\mathbb {Z}}}}_n^d}\overset{d}{=}(Y+\chi _x)_{x\in {{\mathrm{\mathbb {Z}}}}_n^d}. \end{aligned}$$

In particular, $e_n(\cdot )$ admits the representation

$$\begin{aligned} (e_n(x))_{x\in {{\mathrm{\mathbb {Z}}}}_n^d} \overset{d}{=}\left( \chi _x- \min _{z\in {{\mathrm{\mathbb {Z}}}}_n^d} \chi _z\right) _{x\in {{\mathrm{\mathbb {Z}}}}_n^d}. \end{aligned}$$

This decomposition is similar in spirit to the one in the proof of Levine et al. [17, Proposition 1.3], but we stress that the random fields we find are different. The proof of the above Proposition can be found in Sect. 3.1. As a consequence, to achieve Theorem 1 it will suffice to determine the scaling limit of the $\chi$ field, because test functions have zero average, and hence we can get rid of the minimum appearing in the odometer representation. We will therefore show

1. $\left( {\mathcal L}(\Xi _n)\right) _{n\in {{\mathrm{\mathbb {N}}}}}$ is tight in the space ${{\mathcal {H}}}_{-\epsilon }(\mathbb {T}^d)$ where $-\epsilon <-\frac{d}{2}$.
2. From the above tightness result, there exists a subsequential scaling limit $\Xi =\lim _{k\rightarrow +\infty }\Xi _{n_k}$ for the convergence in law in the space ${{\mathcal {H}}}_{-\epsilon }$. The proof is complete once we show this limit is unique: by Ledoux and Talagrand [14, Section 2.1], it suffices to prove that, for all mean-zero test functions $u\in C^\infty (\mathbb {T}^d)$,
  $$\begin{aligned} \lim _{n\rightarrow +\infty }\mathsf {E}\left[ \exp \left( \iota \left\langle \Xi _n,\,u\right\rangle \right) \right] =\Phi (u), \end{aligned}$$
  where the RHS is the characteristic function of $\Xi$. We will calculate the limit of the second moment of $\left\langle \Xi _n,\,u\right\rangle$ directly in $d\le 3$ and through a mollifying procedure in $d\ge 4$.

This will conclude the proof. Since the “finite dimensional” convergence is somewhat more interesting, we will defer the tightness proof to Sect. 4.2 and show (P2) in Sect. 4.1.

*A note on test functions* By the above construction, the set of test functions we will consider is the set of smooth functions $C^\infty (\mathbb T^d)$ with zero mean. We need to stress at this juncture an important remark: $C(\mathbb {T}^d)$ does *not* correspond to the class of continuous functions on $[-\frac{1}{2}, \,\frac{1}{2})^d$, but only to functions which remain continuous on ${{\mathrm{\mathbb {R}}}}^d$ when extended by periodicity. Similar comments apply to $C^{\infty }(\mathbb {T}^d)$ functions. See also Stein and Weiss [26, Section 1, Chapter VII] for further discussions. Therefore, when we consider $u: {{\mathrm{\mathbb {R}}}}^d \rightarrow {{\mathrm{\mathbb {R}}}}$ which is periodic and belongs to $C^\infty$, we consider its restriction to $[-\frac{1}{2}, \,\frac{1}{2})^d$ while computing its integral on $\mathbb {T}^d$.

## Auxiliary results

In this section we provide a proof of Proposition 4. The result helps us tackle the singularity arising from the zero eigenvalue of $\Delta _g$ and will also reduce the determination of the scaling limit to finding the scaling limit of $(\chi _x)_{x\in {{\mathrm{\mathbb {Z}}}}_n^d}$.

### Proof of Proposition 4

#### Proof

First, observe that, by Parseval’s identity on the discrete torus, we can write the covariance of the Gaussian field $(\eta (x))_{x\in {{\mathrm{\mathbb {Z}}}}_n^d}$ as

3.1 $$\begin{aligned} \mathsf {E}\left[ \eta (x) \eta (y)\right]&=(2d)^{-2}\sum _{z\in {{\mathrm{\mathbb {Z}}}}_n^d}g(z,\,x)g(z,\,y)\nonumber \\&=(2d)^{-2}n^{d}\widehat{g_x}(0)\widehat{g_y}(0)+(2d)^{-2}n^{d}\sum _{z\in {{\mathrm{\mathbb {Z}}}}_n^d{\setminus }\{0\}}\widehat{g_x}(z)\overline{\widehat{g_y}(z)}. \end{aligned}$$

First observe that using the description of *g*(*x*, *y*) in terms of the simple random walk $(S_m)_{m\ge 0}$ on ${{\mathrm{\mathbb {Z}}}}_n^d$ we derive

3.2 $$\begin{aligned} \widehat{g_x}(0)&= n^{-d}\sum _{y\in {{\mathrm{\mathbb {Z}}}}_n^d} g_x(y)=n^{-2d}\sum _{y\in {{\mathrm{\mathbb {Z}}}}_n^d} \sum _{z\in {{\mathrm{\mathbb {Z}}}}_n^d}\sum _{m\ge 0} \mathsf P_x( S_m=y, m< \tau _z) \nonumber \\&= n^{-2d}\sum _{z\in {{\mathrm{\mathbb {Z}}}}_n^d} \sum _{y\in {{\mathrm{\mathbb {Z}}}}_n^d{\setminus }\{z\}} \sum _{m\ge 0}\mathsf P_x(S_m=y, m< \tau _z)\nonumber \\&= n^{-2d} \sum _{z\in {{\mathrm{\mathbb {Z}}}}_n^d}\sum _{m\ge 0} \mathsf P_x(\tau _z>m)= n^{-2d} \sum _{z\in {{\mathrm{\mathbb {Z}}}}_n^d}\mathsf {E}_x[ \tau _z]. \end{aligned}$$

One can notice that $\widehat{g_x}(0)$ is independent of *x* by translation invariance. Hence we get that the first term in the left-hand side of (3.1) is a constant equal to $(2d)^{-2}n^d L^2$ having set $L:=n^{-2d}\sum _{q\in {{\mathrm{\mathbb {Z}}}}_n^d}\mathsf E_x[\tau _q]$. As for the contribution from other sites,

$$\begin{aligned} (2d)^{-2}n^{d}\sum _{z\in {{\mathrm{\mathbb {Z}}}}_n^d{\setminus }\{0\}}\widehat{g_x}(z)\overline{\widehat{g_y}(z)}&{\mathop {=}\limits ^{(2.2)}}n^{-d}\sum _{z\in {{\mathrm{\mathbb {Z}}}}_n^d{\setminus }\{0\}}\frac{\exp \left( -2\pi \iota x\cdot \frac{z}{n}\right) \exp \left( 2\pi \iota y\cdot \frac{z}{n}\right) }{|\lambda _z|^2}. \end{aligned}$$

Define a centered Gaussian field $(\chi _x)_{x\in {{\mathrm{\mathbb {Z}}}}_n^d}$ with covariance given by

3.3 $$\begin{aligned} H(x,y)=\frac{n^{-d}}{16} \sum _{z\in {{\mathrm{\mathbb {Z}}}}_n^d{\setminus }\{0\}} \frac{\exp (2\pi \iota (y-x)\cdot \frac{z}{n})}{\left( \sum _{i=1}^d \sin ^2\left( \pi \frac{z_i}{n}\right) \right) ^2}. \end{aligned}$$

The field associated to *H* is well-defined and in fact *H* is positive definite. To see this, given a function $c:{{\mathrm{\mathbb {Z}}}}_n^d\rightarrow \mathbb C$ one has that $\sum _{x,y\in {{\mathrm{\mathbb {Z}}}}_n^d} H(x,y) c(x)\overline{c(y)} \ge 0$. Indeed,

$$\begin{aligned} \sum _{x,y\in {{\mathrm{\mathbb {Z}}}}_n^d} H(x,y) c(x)\overline{c(y)}&=\frac{n^{-d}}{16}\sum _{x,y\in {{\mathrm{\mathbb {Z}}}}_n^d}\sum _{z\in {{\mathrm{\mathbb {Z}}}}_n^d{\setminus }\{0\}} \frac{\exp (2\pi (y-x)\cdot \frac{z}{n})}{\left( \sum _{i=1}^d \sin ^2\left( \pi \frac{z_i}{n}\right) \right) ^2}c(x)\overline{c(y)}\\&=\frac{n^{-d}}{16}\sum _{z\in {{\mathrm{\mathbb {Z}}}}_n^d{\setminus }\{0\}} d(z)\overline{d(z)}\ge 0, \end{aligned}$$

where $d(z):=\sum _{x\in {{\mathrm{\mathbb {Z}}}}_n^d} {\exp (-2\pi \iota x\cdot \frac{z}{n})}{{\left( \sum _{i=1}^d \sin ^2(\pi \frac{z_i}{n})\right) ^{-1}}}c(x).$ Hence it turns out that $(\eta (x))_{x\in {{\mathrm{\mathbb {Z}}}}_n^d}$ has the same distribution as $(Y+\chi _x)_{x\in {{\mathrm{\mathbb {Z}}}}_n^d}$ where *Y* is a Gaussian random variable with mean zero and variance $(2d)^{-2} n^dL^2$ independent of the field $\chi$. To conclude, note that the odometer function satisfies $e_n(x){\mathop {=}\limits ^{d}} \eta (x)-\min _{z\in {{\mathrm{\mathbb {Z}}}}_n^d} \eta (z)\overset{d}{=} \chi _x-\min _{z\in {{\mathrm{\mathbb {Z}}}}_n^d} \chi _z$. $\square$

## Proof of Theorem 1

We recall that it will suffice to prove the two properties (P1) and (P2) to achieve the Theorem. We first use to our advantage the fact that the test functions we consider have zero average, hence we can get rid of the minimum term which appears in the definition of the odometer. Let us recall the field in (1.3)

$$\begin{aligned} \Xi _n(\cdot )=4\pi ^2\sum _{z\in \mathbb {T}^d_n}n^{\frac{d-4}{2}}e_n({nz}){{\mathrm{\mathbb {1}}}}_{B\left( z,\,\frac{1}{2n}\right) }(\cdot ). \end{aligned}$$

We define a linear functional on $C^\infty (\mathbb {T}^d)$ by setting

$$\begin{aligned} \left\langle \Xi _n,\,u \right\rangle :=\int _{\mathbb {T}^d}\left( 4\pi ^2 n^{\frac{d-4}{2}}\sum _{z\in \mathbb {T}^d_n}{{\mathrm{\mathbb {1}}}}_{B\left( z,\,\frac{1}{2n}\right) }(x)e_n({nz})\right) u(x){{\mathrm{d}}}x. \end{aligned}$$

However using Proposition 4, and the fact that *u* has zero mean, one sees that

$$\begin{aligned} \left\langle \Xi _n,\,u \right\rangle&=4\pi ^2\sum _{z\in \mathbb {T}_n^d} n^{\frac{d-4}{2}}\chi _{nz}\int _{B(z,\frac{1}{2n})} u(x){{\mathrm{d}}}x\\&\quad -4\pi ^2\sum _{z\in \mathbb {T}_n^d} n^{\frac{d-4}{2}}\left( \min _{w\in {{\mathrm{\mathbb {Z}}}}_n^d}\chi _{w}\right) \int _{B(z,\frac{1}{2n})} u(x){{\mathrm{d}}}x\\&=4\pi ^2\sum _{z\in \mathbb {T}_n^d} n^{\frac{d-4}{2}}\chi _{nz}\int _{B(z,\frac{1}{2n})} u(x){{\mathrm{d}}}x=\left\langle \Xi '_n,\,u\right\rangle \end{aligned}$$

letting

$$\begin{aligned} \Xi _n'(\cdot ):=4\pi ^2\sum _{z\in \mathbb {T}^d_n}n^{\frac{d-4}{2}}\chi _{nz}{{\mathrm{\mathbb {1}}}}_{B\left( z,\,\frac{1}{2n}\right) }(\cdot ) \end{aligned}$$

By the theory of Gaussian Hilbert spaces of Sect. 2.2, $\Xi _n=\Xi _n'$ in distribution. Hence in the sequel we will, with a slight abuse of notation, consider $\Xi _n'$ but denote it simply as $\Xi _n$, since the law of the two fields is the same. We are now ready to begin with (P2).

### Proof of (P2)

*Overview of the proof* We have just seen that

$$\begin{aligned} \left\langle \Xi _n,\,u \right\rangle =4\pi ^2\sum _{z\in \mathbb {T}_n^d} n^{\frac{d-4}{2}}\chi _{nz}\int _{B(z,\frac{1}{2n})} u(x){{\mathrm{d}}}x. \end{aligned}$$

We now replace the integral over the ball above by the value at its center and gather the remaining error term. More precisely we get

$$\begin{aligned}&4\pi ^2\sum _{z\in \mathbb {T}_n^d} n^{\frac{d-4}{2}}\chi _{nz}\int _{B(z,\frac{1}{2n})} u(x){{\mathrm{d}}}x=4\pi ^2\sum _{z\in \mathbb {T}_n^d} n^{\frac{d-4}{2}}\chi _{nz}n^{-d}\int _{B(z,\frac{1}{2n})} n^d u(x){{\mathrm{d}}}x\\&\quad =4\pi ^2\sum _{z\in \mathbb {T}_n^d} n^{\frac{d-4}{2}}\chi _{nz}n^{-d}u(z) \\&\qquad + 4\pi ^2\sum _{z\in \mathbb {T}_n^d} n^{\frac{d-4}{2}}\chi _{nz}n^{-d}\left( \int _{B(z,\frac{1}{2n})}n^d u(x){{\mathrm{d}}}x-u(z)\right) \\&\quad =4\pi ^2 n^{-\frac{d+4}{2}}\sum _{z\in \mathbb {T}^d_n}\chi _{nz}u(z)+R_n(u). \end{aligned}$$

Here the remainder $R_n(u)$ is defined by

4.1 $$\begin{aligned} R_n(u)&:=4\pi ^2\sum _{z\in \mathbb {T}_n^d} n^{\frac{d-4}{2}}\chi _{nz}n^{-d}\left( \int _{B(z,\,\frac{1}{2n})}n^d u(x){{\mathrm{d}}}x-u(z)\right) \nonumber \\&=4\pi ^2 n^{-\frac{d+4}{2}}\sum _{z\in \mathbb {T}_n^d}\chi _{nz} K_n(z) \end{aligned}$$

where using that the volume of $B(z, \frac{1}{2n})$ is $n^{-d}$ we have

4.2 $$\begin{aligned} K_n(z):=\int _{B(z,\,\frac{1}{2n})}n^d u(x){{\mathrm{d}}}x-u(z)=n^d\left[ \int _{B(z,\,\frac{1}{2n})}\left( u(x)-u(z)\right) {{\mathrm{d}}}x\right] . \end{aligned}$$

We observe that using the above decomposition one can split the variance of $\left\langle \Xi _n,\,u\right\rangle$ as

$$\begin{aligned} \mathsf {E}\left[ \left\langle \Xi _n,\,u\right\rangle ^2 \right]&=16\pi ^4 n^{-(d+4)}\sum _{z,\,z'\in \mathbb {T}_n^d} u(z)u(z') \mathsf {E}[ \chi _{nz}\chi _{nz'}]+\mathsf {E}\left[ R_n(u)^2\right] \\&\quad +4\pi ^2\mathsf {E}\left[ n^{-\frac{d+4}{2}}\sum _{z\in \mathbb {T}_n^d} u(z)\chi _{nz}R_n(u)\right] . \end{aligned}$$

To deal with the convergence of the above terms we need two propositions. The first one shows that the first term yields the required limiting variance.

#### Proposition 5

In the notation of this Section,

$$\begin{aligned}&16\pi ^4\lim _{n\rightarrow +\infty }n^{-(d+4)}\sum _{z,\,z'\in \mathbb {T}_n^d} u(z)u(z') \mathsf {E}[ \chi _{nz}\chi _{nz'}]\\&\quad =16\pi ^4\lim _{n\rightarrow +\infty }n^{-(d+4)}\sum _{z,\,z'\in \mathbb {T}_n^d} u(z)u(z') H\left( {nz},\,{nz'}\right) \\&\quad =\Vert u\Vert ^2_{{-1}}. \end{aligned}$$

The second Proposition says the remainder term is small.

#### Proposition 6

In the notations of this Section, $\lim _{n\rightarrow +\infty }R_n(u)=0$ in $L^2$.

Then an application of the Cauchy-Schwarz inequality will allow us to deduce that

$$\begin{aligned} \lim _{n\rightarrow +\infty }\mathsf {E}\left[ \left\langle \Xi _n,\,u\right\rangle ^2 \right] =\Vert u\Vert ^2_{{-1}} \end{aligned}$$

and the condition (P2) will be ensured. We give the proof of Proposition 5, which is the core of our argument, in Sect. 4.1.1 and of Proposition 6 in Sect. 4.1.2.

#### Proof of Proposition 5

Before we begin our proof we would like to prove a bound which would be crucial in estimating the eigenvalues of the Laplacian on the discrete torus. This lemma will be used later for other parts of the proof too.

##### Lemma 7

There exists $c>0$ such that for all $n\in {{\mathrm{\mathbb {N}}}}$ and $w\in {{\mathrm{\mathbb {Z}}}}_n^d{\setminus }\{0\}$ we have

4.3 $$\begin{aligned} \frac{1}{\Vert \pi w\Vert ^4}\le n^{-4}\left( \sum _{i=1}^d \sin ^2\left( \frac{\pi w_i}{n}\right) \right) ^{-2}\le \left( \frac{1}{\Vert \pi w\Vert ^2}+\frac{c}{n^{2}}\right) ^2 \end{aligned}$$

##### Proof

We consider

$$\begin{aligned} \sum _{i=1}^d n^2\sin ^2\left( \frac{\pi w_i}{n}\right) =\sum _{i=1}^d w_i^2\pi ^2\left( \frac{\sin \left( \theta _i^n\right) }{\theta _i^n}\right) ^2 \end{aligned}$$

with $\theta _i^n:=\pi { w_i}{n^{-1}}\in \left[ -{\pi }/{2},\,{\pi }/{2}\right] {\setminus }\{0\}.$ This gives the left-hand side of (4.3). Moreover

$$\begin{aligned} \left\| \pi w\right\| ^2-\sum _{i=1}^d n^2\sin ^2\left( \frac{\pi w_i}{n}\right) =\sum _{i=1}^d w_i^2\pi ^2\left( 1-\left( \frac{\sin \left( \theta _i^n\right) }{\theta _i^n}\right) ^2\right) \le C\Vert w\Vert ^4 n^{-2} \end{aligned}$$

because $0\le 1-{\sin ^2(x)}{x^{-2}}\le C\,x^{2}$ for some $C>0$. In this way

4.4 $$\begin{aligned}&\frac{1}{\sum _{i=1}^d n^2\sin ^2\left( \frac{\pi w_i}{n}\right) }-\frac{1}{\Vert \pi w\Vert ^2}=\frac{\Vert \pi w\Vert ^2-\sum _{i=1}^d n^2\sin ^2\left( \frac{\pi w_i}{n}\right) }{\sum _{i=1}^d n^2\sin ^2\left( \frac{\pi w_i}{n}\right) \Vert \pi w\Vert ^2}\nonumber \\&\quad \le \frac{C\Vert w\Vert ^4n^{-2}}{\sum _{i=1}^d n^2\sin ^2\left( \frac{\pi w_i}{n}\right) \Vert \pi w\Vert ^2}. \end{aligned}$$

Considering that, for $x\in \left[ -{\pi }/{2},\,{\pi }/{2}\right]$, ${\sin ^2(x)}{x^{-2}}\in \left[ {4}/{\pi ^2},\,1\right]$, one gets that

4.5 $$\begin{aligned} \sum _{i=1}^d n^2\sin ^2\left( \frac{\pi w_i}{n}\right) \ge {4}\Vert w\Vert ^2 \end{aligned}$$

which plugged into (4.4) gives that

$$\begin{aligned} \frac{1}{\sum _{i=1}^d n^2\sin ^2\left( \frac{\pi w_i}{n}\right) }-\frac{1}{\Vert \pi w\Vert ^2}\le C{n^{-2}} \end{aligned}$$

for $C>0$, thus (4.3) is proven. $\square$

##### Remark 2

The equation (4.5) is not enough to obtain sharp asymptotics for $\sum _{i=1}^d n^2 \sin ^2\left( \pi {w_i}/{n}\right)$ as $n\rightarrow \infty$. On the other hand, we will use it in the sequel while looking for a uniform lower bound for the same quantity for all $w\ne 0$.

We begin with the proof of Proposition 5. Let $u : \mathbb {T}^d\rightarrow {{\mathrm{\mathbb {R}}}}$ be a smooth function with zero mean. Define $u_n:{{\mathrm{\mathbb {Z}}}}_n^d\rightarrow {{\mathrm{\mathbb {R}}}}$ as $u_n(z):= u(\frac{z}{n})$. Note that

4.6 $$\begin{aligned}&16\pi ^4 n^{-2d} n^{d-4}\sum _{z,\,z'\in \mathbb {T}_n^d} u(z)u(z') \mathsf {E}[ \chi _{nz}\chi _{nz'}]\nonumber \\&\quad = 16\pi ^4 n^{-2d}n^{d-4} \sum _{z,\,z'\in {{\mathrm{\mathbb {Z}}}}_n^d} u(z) u(z') H(nz, nz')\nonumber \\&\quad =\pi ^4 n^{-2d}n^{-4} \sum _{z,\,z'\in \mathbb {T}_n^d} u(z) u(z') \sum _{w\in {{\mathrm{\mathbb {Z}}}}_n^d{\setminus }\{0\}}\frac{\exp (2\pi \iota (z-z')\cdot w)}{\left( \sum _{i=1}^d\sin ^2\left( \frac{\pi w_i}{n}\right) \right) ^2}. \end{aligned}$$

To show the above expression converges it is enough to consider the convergence of

4.7 $$\begin{aligned} n^{-2d}\sum _{z,\,z'\in \mathbb {T}_n^d} u(z) u(z') \sum _{w\in {{\mathrm{\mathbb {Z}}}}_n^d{\setminus }\{0\}}\frac{\exp (2\pi \iota (z-z')\cdot w)}{\Vert w\Vert ^4}. \end{aligned}$$

This can be justified by showing that (4.6) can be bounded above and below appropriately by (4.7). Now observing that

4.8 $$\begin{aligned} n^{-2d}\sum _{z,\,z'\in \mathbb {T}_n^d} u(z) u(z') \exp (2\pi \iota (z-z')\cdot w)= \left| \widehat{ u_n}(w)\right| ^2\ge 0 \end{aligned}$$

the lower bound of (4.3) immediately gives

$$\begin{aligned}&\pi ^4 n^{-2d-4} \sum _{z,\,z'\in \mathbb {T}_n^d} u(z) u(z') \sum _{w\in {{\mathrm{\mathbb {Z}}}}_n^d{\setminus }\{0\}}\frac{\exp (2\pi \iota (z-z')\cdot w)}{\left( \sum _{i=1}^d\sin ^2\left( \frac{\pi w_i}{n}\right) \right) ^2}\\&\quad \ge n^{-2d} \sum _{z,\,z'\in \mathbb {T}_n^d} u(z) u(z') \sum _{w\in {{\mathrm{\mathbb {Z}}}}_n^d{\setminus }\{0\}}\frac{\exp (2\pi \iota (z-z')\cdot w)}{\Vert w\Vert ^4}. \end{aligned}$$

For the upper bound, using the bound in (4.3) we get

$$\begin{aligned}&\pi ^4 n^{-2d-4} \sum _{z,\,z'\in \mathbb {T}_n^d} u(z) u(z') \sum _{w\in {{\mathrm{\mathbb {Z}}}}_n^d{\setminus }\{0\}}\frac{\exp (2\pi \iota (z-z')\cdot w)}{\left( \sum _{i=1}^d\sin ^2\left( \frac{\pi w_i}{n}\right) \right) ^2}\\&\quad \le \pi ^4 n^{-2d} \sum _{z,\,z'\in \mathbb {T}_n^d} u(z) u(z') \sum _{w\in {{\mathrm{\mathbb {Z}}}}_n^d{\setminus }\{0\}}{\exp (2\pi \iota (z-z')\cdot w)}\left( \frac{1}{\Vert \pi w\Vert ^{2}}+\frac{c}{n^{2}}\right) ^2. \end{aligned}$$

Now we expand the square: the first term gives the correct upper bound as in (4.7) and the other two terms are negligible. In fact we show firstly that

$$\begin{aligned} \lim _{n\rightarrow +\infty } c n^{-2d}n^{-2} \sum _{z,\,z'\in \mathbb {T}_n^d} u(z) u(z') \sum _{w\in {{\mathrm{\mathbb {Z}}}}_n^d{\setminus }\{0\}}\frac{\exp (2\pi \iota (z-z')\cdot w)}{\Vert w\Vert ^2}=0. \end{aligned}$$

Using (4.8) and Parseval’s identity we get

$$\begin{aligned}&c n^{-2d}n^{-2} \sum _{z,\,z'\in \mathbb {T}_n^d} u(z) u(z') \sum _{w\in {{\mathrm{\mathbb {Z}}}}_n^d{\setminus }\{0\}}\frac{\exp (2\pi \iota (z-z')\cdot w)}{\Vert w\Vert ^2}\\&\quad = c n^{-2}\sum _{w\in {{\mathrm{\mathbb {Z}}}}_n^d{\setminus }\{0\}} \frac{1}{\Vert w\Vert ^2} \left| \widehat{u_n}(w)\right| ^2\\&\quad \overset{\Vert w\Vert \ge 1}{\le }c n^{-2}\sum _{w\in {{\mathrm{\mathbb {Z}}}}_n^d{\setminus }\{0\}} |\widehat{u_n}(w)|^2\le c n^{-2}\sum _{w\in {{\mathrm{\mathbb {Z}}}}_n^d} \left| \widehat{u_n}(w)\right| ^2\\&\quad = c n^{-2}n^{-d} \sum _{w\in {{\mathrm{\mathbb {Z}}}}_n^d} \left| u\left( \frac{w}{n}\right) \right| ^2=c n^{-2}\left( n^{-d}\sum _{w\in \mathbb {T}_n^d} |u(w)|^2\right) . \end{aligned}$$

Since $n^{-d}\sum _{w\in \mathbb {T}_n^d} |u(w)|^2\rightarrow \int _{\mathbb {T}^d} |u(w)|^2{{\mathrm{d}}}w<+\infty$ we get that the second term converges to zero. Note that the same computation shows

$$\begin{aligned} n^{-2d} n^{-4}\sum _{z,\,z'\in \mathbb {T}_n^d} u(z) u(z') \sum _{w\in {{\mathrm{\mathbb {Z}}}}_n^d{\setminus }\{0\}}\exp (2\pi \iota (z-z')\cdot w)\le n^{-4}\left( n^{-d}\sum _{w\in \mathbb {T}_n^d} |u(w)|^2\right) , \end{aligned}$$

which again goes to zero as $n\rightarrow + \infty$. So this shows that we can from now on concentrate on showing the convergence of (4.7). We split now our proof, according to whether $d\le 3$ or $d\ge 4$.

*The case* $d\le 3$ In the first case, the argument is more straightforward: we rewrite

$$\begin{aligned} (4.7)= \sum _{w\in {{\mathrm{\mathbb {Z}}}}^d{\setminus }\{0\}}\Vert w\Vert ^{-4}{{\mathrm{\mathbb {1}}}}_{w\in {{\mathrm{\mathbb {Z}}}}_n^d}\sum _{z\in \mathbb {T}_n^d} n^{-d}u(z)\exp (2\pi \iota z\cdot w) \sum _{z'\in \mathbb {T}_n^d}n^{-d}u(z')\exp (-2\pi \iota z'\cdot w). \end{aligned}$$

Since $\sum _{z\in \mathbb {T}_n^d} n^{-d}u(z)\exp (2\pi \iota z\cdot w)$ is bounded above uniformly in *n*, and $\sum _{w\in {{\mathrm{\mathbb {Z}}}}^d{\setminus }\{0\}}\Vert w\Vert ^{-4}<+\infty$ in $d<4$, we can apply the dominated converge theorem and obtain

$$\begin{aligned} \lim _{n\rightarrow +\infty }(4.7)=\sum _{w\in {{\mathrm{\mathbb {Z}}}}^d{\setminus }\{0\}}\Vert w\Vert ^{-4}\left| {\widehat{u}}(w)\right| ^2=\Vert u\Vert ^2_{-1} \end{aligned}$$

which concludes the proof of (P2) for $d\le 3$.

*The case* $d\ge 4$ Here it is necessary to think of another strategy since $\sum _{w\in {{\mathrm{\mathbb {Z}}}}^d}\Vert w\Vert ^{-4}$ is not finite. Let $\phi \in {\mathcal S}({{\mathrm{\mathbb {R}}}}^d)$, the Schwartz space, be a mollifier supported on $[-\frac{1}{2}, \,\frac{1}{2})^d$ with $\int _{{{\mathrm{\mathbb {R}}}}^d}\phi (x){{\mathrm{d}}}x=1$ and let $\phi _\kappa (x):= \kappa ^{-d}\phi (\frac{x}{\kappa })$ for $\kappa >0$. It is a classical result [22, Theorem 7.22] that for $\delta =0,\,1,\,2\,\ldots$ there exists $A>0$ (depending on $\kappa$ and $\delta$) such that

4.9 $$\begin{aligned} \left| {\widehat{\phi _\kappa }}(w)\right| \le A\left( 1+\Vert w\Vert \right) ^{-\delta } . \end{aligned}$$

Now to show the convergence of (4.7) is equivalent to considering

$$\begin{aligned} \lim _{\kappa \rightarrow 0}\lim _{n\rightarrow +\infty }n^{-2d}\sum _{z,\,z'\in \mathbb {T}_n^d} u(z) u(z') \sum _{w\in {{\mathrm{\mathbb {Z}}}}_n^d{\setminus }\{0\}}{\widehat{\phi _\kappa }}(w)\frac{\exp (2\pi \iota (z-z')\cdot w)}{\Vert w\Vert ^4} \end{aligned}$$

since we claim that

4.10 $$\begin{aligned} \lim _{\kappa \rightarrow 0}\limsup _{n\rightarrow +\infty }n^{-2d}\sum _{z,\,z'\in \mathbb {T}_n^d} u(z) u(z') \sum _{w\in {{\mathrm{\mathbb {Z}}}}_n^d{\setminus }\{0\}}\left( {\widehat{\phi _\kappa }}(w)-1\right) \frac{\exp (2\pi \iota (z-z')\cdot w)}{\Vert w\Vert ^4}=0. \end{aligned}$$

Indeed, using the fact that $\int _{{{\mathrm{\mathbb {R}}}}^d} \phi _\kappa (x){{\mathrm{d}}}x=1$ we have

$$\begin{aligned} \left| {\widehat{\phi _\kappa }}(w)-1\right| \le \int _{{{\mathrm{\mathbb {R}}}}^d} \phi _\kappa (y)\left| {{\mathrm{e}}}^{2\pi \iota y\cdot w}-1\right| {{\mathrm{d}}}y. \end{aligned}$$

Exploiting the fact that $|\exp (2\pi \iota x)-1|^2= 4\sin ^2(\pi x)$ and $|\sin (x)|\le |x|$ we obtain

4.11 $$\begin{aligned} \left| {\widehat{\phi _\kappa }}(w)-1\right| \le C\kappa \Vert w\Vert \int _{{{\mathrm{\mathbb {R}}}}^d} \Vert y\Vert \phi (y) {{\mathrm{d}}}y\le C \kappa \Vert w\Vert \end{aligned}$$

due to the fact that $\phi$ is supported on $[-\frac{1}{2},\,\frac{1}{2})^d$. Recalling $u_n(z)=u(\frac{z}{n})$ and plugging the estimate (4.11) in (4.10) we get that

4.12 $$\begin{aligned}&\left| n^{-2d}\sum _{w\in {{\mathrm{\mathbb {Z}}}}_n^d{\setminus }\{0\}}\frac{{\widehat{\phi _\kappa }}(w)-1}{\Vert w\Vert ^4}\sum _{z,\,z'\in \mathbb {T}_n^d} u(z) u(z') \exp (2\pi \iota (z-z')\cdot w)\right| \nonumber \\&\quad \le C\kappa \sum _{w\in {{\mathrm{\mathbb {Z}}}}_n^d{\setminus } \{0\}} \Vert w\Vert ^{-3} \left| \widehat{u_n} (w)\right| ^2. \end{aligned}$$

Using $\Vert w\Vert \ge 1$ we have

$$\begin{aligned}&\sum _{w\in {{\mathrm{\mathbb {Z}}}}_n^d{\setminus } \{0\}} \Vert w\Vert ^{-3} \left| \widehat{u_{n}} (w)\right| ^2\le \sum _{w\in {{\mathrm{\mathbb {Z}}}}_n^d{\setminus } \{0\}} \left| \widehat{u_{n}} (w)\right| ^2\le \sum _{w\in {{\mathrm{\mathbb {Z}}}}_n^d} \left| \widehat{u_{n}} (w)\right| ^2\\&\quad =n^{-d} \sum _{w\in {{\mathrm{\mathbb {Z}}}}_n^d} \left| u\left( \frac{w}{n}\right) \right| ^2 = n^{-d}\sum _{w\in \mathbb {T}_n^d} |u(w)|^2 \end{aligned}$$

where we have used Parseval’s identity. We observe then that

$$\begin{aligned}&\limsup _{n\rightarrow +\infty }\left| n^{-2d}\sum _{w\in {{\mathrm{\mathbb {Z}}}}_n^d {\setminus }\{0\}}\frac{{\widehat{\phi _\kappa }}(w)-1}{\Vert w\Vert ^4} \sum _{z,\,z'\in \mathbb {T}_n^d} u(z) u(z') \exp (2\pi \iota (z-z')\cdot w)\right| \\&\quad \le C\kappa \Vert u\Vert _{L^2(\mathbb {T}^d)}^2<+\infty . \end{aligned}$$

Taking the limit $\kappa \rightarrow 0$ in the previous expression we deduce the claim (4.10). Now we have to derive the limit of the following expression:

4.13 $$\begin{aligned} n^{-2d}\sum _{z,\,z'\in \mathbb {T}_n^d} u(z) u(z') \sum _{w\in {{\mathrm{\mathbb {Z}}}}_n^d{\setminus }\{0\}}{\widehat{\phi _\kappa }}(w)\frac{\exp (2\pi \iota (z-z')\cdot w)}{\Vert w\Vert ^4}. \end{aligned}$$

Since ${\widehat{\phi _\kappa }}$ has a fast decay at infinity, and

$$\begin{aligned} \lim _{n\rightarrow +\infty }n^{-d}\sum _{z\in \mathbb {T}_n^d} u(z) \exp (2\pi \iota z\cdot w)={\widehat{u}}(w) \end{aligned}$$

we can apply the dominated convergence theorem to obtain

$$\begin{aligned}&\lim _{n\rightarrow +\infty }n^{-2d}\sum _{z,\,z'\in \mathbb {T}_n^d} u(z) u(z') \sum _{w\in {{\mathrm{\mathbb {Z}}}}_n^d{\setminus }\{0\}}{\widehat{\phi _\kappa }}(w)\frac{\exp (2\pi \iota (z-z')\cdot w)}{\Vert w\Vert ^4}\\&\quad =\sum _{w\in {{\mathrm{\mathbb {Z}}}}^d{\setminus }\{0\}}{\widehat{\phi _\kappa }}(w)\frac{\left| \widehat{u}(w)\right| ^2}{\Vert w\Vert ^4}. \end{aligned}$$

The bound $|{\widehat{\phi _\kappa }}(\cdot )|\le 1$ can be used to obtain a bound uniform in $\kappa$ on the right-hand side of the above expression: consequently we apply the dominated convergence letting $\kappa \rightarrow 0$ to achieve

$$\begin{aligned}&\lim _{\kappa \rightarrow 0}\lim _{n\rightarrow +\infty }n^{-2d}\sum _{z,\,z'\in \mathbb {T}_n^d} u(z) u(z') \sum _{w\in {{\mathrm{\mathbb {Z}}}}_n^d{\setminus }\{0\}}{\widehat{\phi _\kappa }}(w)\frac{\exp (2\pi \iota (z-z')\cdot w)}{\Vert w\Vert ^4}\\&\quad =\sum _{w\in {{\mathrm{\mathbb {Z}}}}^d{\setminus }\{0\}}\frac{\left| \widehat{u}(w)\right| ^2}{\Vert w\Vert ^4}=\Vert u\Vert ^2_{-1}. \end{aligned}$$

This concludes the proof of Proposition 5.

#### Proof on the remainder: Proposition 6

We owe the reader now the last proofs on $R_n$ (see (4.1)). First we state the following

##### Lemma 8

There exists a constant $C>0$ such that $\sup _{z\in \mathbb {T}^d}|K_n(z)|\le Cn^{-1}$.

##### Proof

Using the mean value theorem as $u\in C^\infty (\mathbb {T}^d)$ we get that, for some $c\in (0,1)$,

$$\begin{aligned} |K_n(z)|&\le n^d\int _{B\left( z,\frac{1}{2n}\right) } |u(x)-u(z)|{{\mathrm{d}}}x\\&\le n^d\int _{B\left( z,\frac{1}{2n}\right) } \Vert \nabla u(cx+(1-c)z) \Vert \,\Vert z-x\Vert {{\mathrm{d}}}x\\&\le C\frac{ n^d}{2n} \int _{B\left( z, \frac{1}{2n}\right) } \Vert \nabla u(cx+(1-c)z) \Vert {{\mathrm{d}}}x\le C \frac{\Vert \nabla u\Vert _{L^\infty (\mathbb {T}^d)}}{n}. \end{aligned}$$

Since $\Vert \nabla u\Vert _{L^\infty (\mathbb {T}^d)}<+\infty$ the claim follows. $\square$

We reprise now the proof on the limit of $R_n(u).$

##### Proof of Proposition 6

We first compute $\mathsf {E}\left[ R_n(u)^2\right]$ obtaining

$$\begin{aligned} \mathsf {E}\left[ R_n(u)^2\right]&=16\pi ^4 n^{-2d}\sum _{z,\,z'\in \mathbb {T}^d_n}n^{d-4}H\left( nz,\,nz'\right) K_n(z)K_n\left( z'\right) \\&{\mathop {\le }\limits ^{(4.5)}}{n^{-2d}} \sum _{z,\,z'\in \mathbb {T}^d_n}\sum _{w\in {{\mathrm{\mathbb {Z}}}}_n^d{\setminus }\{0\}}\frac{\exp (2\pi \iota (z-z')\cdot w)}{\Vert w\Vert ^4}K_n(z)K_n\left( z'\right) \\&\le n^{-2d} \sum _{z,\,z'\in \mathbb {T}^d_n}\sum _{w\in {{\mathrm{\mathbb {Z}}}}_n^d{\setminus }\{0\}}{\exp (2\pi \iota (z-z')\cdot w)}K_n(z)K_n\left( z'\right) \end{aligned}$$

since $\Vert w\Vert \ge 1$. Letting $K'_n(x): =K(\frac{x}{n})$, thanks to Lemma 8 we have that the previous expression is equal to

$$\begin{aligned}&\sum _{w\in {{\mathrm{\mathbb {Z}}}}_n^d{\setminus }\{0\}} \widehat{K'_n}(w)\overline{\widehat{K'_n}(w)} \le \sum _{w\in {{\mathrm{\mathbb {Z}}}}_n^d } \widehat{K'_n}(w)\overline{\widehat{K'_n}(w)} \\&\quad = {n^{-d}} \sum _{w\in {{\mathrm{\mathbb {Z}}}}_n^d } K'_n(w)\overline{K'_n(w)} \le {||K_n||^2_{L^{\infty }(\mathbb {T}^d)}} \le C n^{-2}. \end{aligned}$$

This shows immediately that $R_n(u)$ converges in $L^2$ to 0. $\square$

We are then done with the proof of (P2) on page 7.

### Tightness: proof of (P1)

We proceed to prove tightness. Before that, we must introduce a fundamental result: Rellich’s theorem.

#### Theorem 9

(Rellich’s theorem) If $k_1<k_2$ the inclusion operator $H^{k_2}(\mathbb {T}^d)\hookrightarrow H^{k_1}(\mathbb {T}^d)$ is a compact linear operator. In particular for any radius $R>0$, the closed ball $\overline{B_{\mathcal H_{-\frac{\epsilon }{2}}}(0,\,R)}$ is compact in $\mathcal H_{-\epsilon }$.

*Sketch of the proof * The proof is readily adapted from the one in Roe [21, Theorem 5.8]. Let $\omega >0$ be arbitrarily small. Let *B* be the unit ball of $H^{k_2}(\mathbb {T}^d)$. We quotient then the space $H^{k_2}(\mathbb {T}^d)$ by the subspace $Z:=\left\{ f:\,\widehat{f}(\nu )=0\text { for }\Vert \nu \Vert >N\right\}$ with $N=N(\omega )$ large enough so that $\Vert f\Vert _{{k_1}}<\omega$ for $f\in B\cap Z$. The unitary ball in $H^{k_2}/Z$ is then compact and thus can be covered by finitely many $\omega$-balls, giving a finite $2\omega$-covering of balls for *B* in the $H^{k_1}$-norm as well. This shows the inclusion operator is compact.

We take $k_1:=-\epsilon$ and $k_2:=-\frac{\epsilon }{2}$. By the definitions in Sect. 2.2, there is a Hilbert space isomorphism between $H^{a}(\mathbb {T}^d)$ and ${\mathcal {H}}_{a}(\mathbb {T}^d)$. Applying the above observation, we get the result. $\square$

#### Proof of tightness

Choose $-\epsilon <-\frac{d}{2}$. Observe that

$$\begin{aligned} \Vert \Xi _n\Vert _{L^2(\mathbb {T}^d)}^2=16\pi ^4 n^{d-4}\sum _{x,\,y\in \mathbb {T}_n^d}\left( \chi _{nx}-\min _{w\in {{\mathrm{\mathbb {Z}}}}_n^d}\chi _w\right) \left( \chi _{ny}-\min _{w\in {{\mathrm{\mathbb {Z}}}}_n^d}\chi _w\right) \end{aligned}$$

is a. s. finite, for fixed *n*, being a finite combination of Gaussian variables and their minimum. Hence $\Xi _n\in L^2(\mathbb {T}^d)\subset {\mathcal {H}}_{-\epsilon }(\mathbb {T}^d)$ a. s. By Rellich’s theorem it will suffice to find, for all $\delta >0$, a $R=R(\delta )>0$ such that

$$\begin{aligned} \sup _{n\in {{\mathrm{\mathbb {N}}}}}\mathsf {P}\left( \Vert \Xi _n\Vert _{\mathcal H_{-\frac{\epsilon }{2}}}\ge R\right) \le \delta . \end{aligned}$$

A consequence of Markov’s inequality is that such an $R(\delta )$ can be found as long as we show that for some $C>0$

$$\begin{aligned} \sup _{n\in {{\mathrm{\mathbb {N}}}}}\mathsf {E}\left[ \Vert \Xi _n\Vert _{\mathcal H_{-\frac{\epsilon }{2}}}^2\right] \le C. \end{aligned}$$

Since $\Xi _n\in L^2$, it admits a Fourier series representation $\Xi _n(\vartheta )=\sum _{\nu \in {{\mathrm{\mathbb {Z}}}}^d}\widehat{\Xi _n}(\nu ){\mathbf {e}}_\nu (\vartheta )$ with $\widehat{\Xi _n}(\nu )=(\Xi _n,\,{\mathbf {e}}_\nu )_{L^2(\mathbb {T}^d)}$. Thus we can express

$$\begin{aligned} \Vert \Xi _n\Vert _{{\mathcal {H}}_{-\frac{\epsilon }{2}}}^2&=\sum _{\nu \in {{\mathrm{\mathbb {Z}}}}^d{\setminus }\{0\}} \Vert \nu \Vert ^{-2\epsilon }\left| \widehat{\Xi _n}(\nu )\right| ^2. \end{aligned}$$

Observe that

$$\begin{aligned} \widehat{\Xi _n}(\nu )=\int _{\mathbb {T}^d}\Xi _n(\vartheta ){\mathbf {e}}_{\nu }(\vartheta ){{\mathrm{d}}}\vartheta =4\pi ^2\sum _{x\in \mathbb {T}^d_n}n^{\frac{d-4}{2}}\chi _{nx}\int _{B(x,\,\frac{1}{2n})}{\mathbf {e}}_{\nu }(\vartheta ){{\mathrm{d}}}\vartheta . \end{aligned}$$

This gives

4.14 $$\begin{aligned}&\mathsf {E}\left[ \Vert \Xi _n\Vert _{\mathcal H_{-\frac{\epsilon }{2}}}^2\right] \nonumber \\&\quad =16\pi ^4\sum _{\nu \in {{\mathrm{\mathbb {Z}}}}^d{\setminus }\{0\}}\sum _{x,\,y\in \mathbb {T}^d_n}\Vert \nu \Vert ^{-2\epsilon }n^{d-4}\mathsf {E}\left[ \chi _{nx}\chi _{ny}\right] \int _{B(x,\,\frac{1}{2n})}{\mathbf {e}}_{\nu }(\vartheta ){{\mathrm{d}}}\vartheta \int _{B(y,\,\frac{1}{2n})}\overline{{\mathbf {e}}_{\nu }(\vartheta )}{{\mathrm{d}}}\vartheta \nonumber \\&\quad =16\pi ^4\sum _{\nu \in {{\mathrm{\mathbb {Z}}}}^d{\setminus }\{0\}}\sum _{x,\,y\in \mathbb {T}^d_n}\Vert \nu \Vert ^{-2\epsilon }n^{d-4}H(nx,\,ny)\int _{B(x,\,\frac{1}{2n})}{\mathbf {e}}_{\nu }(\vartheta ){{\mathrm{d}}}\vartheta \int _{B(y,\,\frac{1}{2n})}\overline{{\mathbf {e}}_{\nu }(\vartheta )}{{\mathrm{d}}}\vartheta . \end{aligned}$$

Let us denote by $F_{n,\,\nu }:\mathbb {T}^d_n\rightarrow {{\mathrm{\mathbb {R}}}}$ the function $F_{n,\,\nu }(x):=\int _{B(x,\,\frac{1}{2n})}{\mathbf {e}}_{\nu }(\vartheta ){{\mathrm{d}}}\vartheta$. Since ${\mathbf {e}}_\nu \in L^2(\mathbb {T}^d)$, the Cauchy-Schwarz inequality implies that $F_{n,\nu }\in L^1(\mathbb {T}^d)$.

Assume we can prove

#### Claim 10

There exists $C'>0$ such that

4.15 $$\begin{aligned} \sup _{\nu \in {{\mathrm{\mathbb {Z}}}}^d}\sup _{n\in {{\mathrm{\mathbb {N}}}}}\sum _{x,\, y\in \mathbb {T}^d_n}n^{d-4}H(nx,\,ny)F_{n,\,\nu }(x)\overline{F_{n,\,\nu }(y)}\le C'. \end{aligned}$$

Using the above Claim and $-\epsilon <-\frac{d}{2},$ from (4.14) we get

$$\begin{aligned} \mathsf {E}\left[ \Vert \Xi _n\Vert _{{\mathcal {H}}_{-\frac{\epsilon }{2}}}^2\right] =&16\pi ^4\sum _{\nu \in {{\mathrm{\mathbb {Z}}}}^d{\setminus }\{0\}}\Vert \nu \Vert ^{-2\epsilon }\sum _{x, \, y\in \mathbb {T}^d_n}n^{d-4}H(nx,\,ny)F_{n,\,\nu }(x)\overline{F_{n,\,\nu }(y)}\\&\le C'\sum _{k\ge 1}k^{d-1-2\epsilon }\le C. \end{aligned}$$

This concludes the proof, assuming Claim 10. $\square$

We are then left to show the claim we have made:

#### Proof of Claim 10

First we use the bound (4.5) and the fact that

$$\begin{aligned} \sum _{x,\,y\in \mathbb {T}_n^d} \exp (2\pi \iota (x-y)\cdot w) F_{n,\,\nu }(x) \overline{F_{n,\,\nu }(y)}=\left| \widehat{F_{n,\,\nu }}(w)\right| ^2 n^{2d}\ge 0 \end{aligned}$$

to obtain

4.16 $$\begin{aligned}&\sum _{x,\,y\in \mathbb {T}^d_n}n^{d-4}H(nx,\,ny)F_{n,\,\nu }(x)\overline{F_{n,\,\nu }(y)}\nonumber \\&\quad =\sum _{x,\,y\in \mathbb {T}^d_n}\frac{n^{d-4}n^{-d}}{16}\sum _{w\in {{\mathrm{\mathbb {Z}}}}_n^d{\setminus } \{0\}}\frac{\exp (2\pi \iota (x-y)\cdot w)}{\left( \sum _{i=1}^d\sin ^2\left( \pi \frac{w_i}{n}\right) \right) ^2}F_{n,\,\nu }(x)\overline{F_{n,\,\nu }(y)}\nonumber \\&\quad \overset{(4.5)}{\le }C \sum _{x,\,y\in \mathbb {T}^d_n}\sum _{w\in {{\mathrm{\mathbb {Z}}}}_n^d{\setminus } \{0\}}\frac{\exp (2\pi \iota (x-y)\cdot w)}{\Vert w\Vert ^4}F_{n,\,\nu }(x)\overline{F_{n,\,\nu }(y)} \end{aligned}$$

Choose a mollifier $\phi _\kappa$ as in the previous considerations (see below (6.1)). We rewrite the expression in the right-hand side of (4.16) accordingly as

4.17 $$\begin{aligned}&C\sum _{x,\,y\in \mathbb {T}^d_n}\sum _{w\in {{\mathrm{\mathbb {Z}}}}_n^d{\setminus } \{0\}}{\widehat{\phi _\kappa }}(w)\frac{\exp (2\pi \iota (x-y)\cdot w)}{\Vert w\Vert ^4}F_{n,\,\nu }(x)\overline{F_{n,\,\nu }(y)}\nonumber \\&\quad +C\sum _{x,\,y\in \mathbb {T}^d_n}\sum _{w\in {{\mathrm{\mathbb {Z}}}}_n^d{\setminus } \{0\}}\left( 1-{\widehat{\phi _\kappa }}(w)\right) \frac{\exp (2\pi \iota (x-y)\cdot w)}{\Vert w\Vert ^4}F_{n,\,\nu }(x)\overline{F_{n,\,\nu }(y)}. \end{aligned}$$

First we get a bound for the second term. Denote as $G_{n,\,\nu }:\,{{\mathrm{\mathbb {Z}}}}_n^d\rightarrow {{\mathrm{\mathbb {R}}}}$ the rescaled function $G_{n,\,\nu }(z):= F_{n,\,\nu }(\frac{z}{n})$. Now we have

$$\begin{aligned}&C\sum _{x,\,y\in \mathbb {T}^d_n}\sum _{w\in {{\mathrm{\mathbb {Z}}}}_n^d{\setminus } \{0\}}\left( 1-{\widehat{\phi _\kappa }}(w)\right) \frac{\exp (2\pi \iota (x-y)\cdot w)}{\Vert w\Vert ^4}F_{n,\,\nu }(x)\overline{F_{n,\,\nu }(y)}\\&\quad =C\sum _{w\in {{\mathrm{\mathbb {Z}}}}_n^d{\setminus } \{0\}}\frac{1-{\widehat{\phi _\kappa }}(w)}{\Vert w\Vert ^4}\sum _{x,\,y\in {{\mathrm{\mathbb {Z}}}}^d_n}F_{n,\,\nu }(\frac{x}{n})\overline{F_{n,\,\nu }(\frac{y}{n})}\exp \left( 2\pi \iota (x-y)\cdot \frac{w}{n}\right) \\&\quad =C n^{2d}\sum _{w\in {{\mathrm{\mathbb {Z}}}}_n^d{\setminus } \{0\}}\frac{1-{\widehat{\phi _\kappa }}(w)}{\Vert w\Vert ^4} \widehat{G_{n,\,\nu }}(w) \overline{\widehat{G_{n,\,\nu }}(w)}\overset{(4.11)}{\le }C \kappa n^{2d} \sum _{w\in {{\mathrm{\mathbb {Z}}}}_n^d}\left| \widehat{G_{n,\,\nu }}(w)\right| ^2 \end{aligned}$$

where in the last inequality we have used that $\Vert w\Vert \ge 1$ and $\left| \widehat{G_{n,\,\nu }}(0)\right| ^2\ge 0$. The description of $G_{n,\,\nu }$, the fact that $|F_{n,\,\nu }(w)|\le n^{-d}$ and Parseval give

4.18 $$\begin{aligned} \sum _{w\in {{\mathrm{\mathbb {Z}}}}_n^d}\left| \widehat{G_{n,\,\nu }}(w)\right| ^2&= n^{-d} \sum _{w\in {{\mathrm{\mathbb {Z}}}}_n^d} G_{n,\,\nu }(w)\overline{G_{n,\,\nu }(w)}=n^{-d}\sum _{w\in \mathbb {T}_n^d} F_{n,\,\nu }(w) \overline{F_{n,\,\nu }(w)}\nonumber \\&\le n^{-2d}\sum _{w\in \mathbb {T}_n^d}\int _{B(w,\,\frac{1}{2n})} |{\mathbf {e}}_\nu (\vartheta )|{{\mathrm{d}}}\vartheta =n^{-2d}\int _{\mathbb {T}^d} |{\mathbf {e}}_\nu (\vartheta )|{{\mathrm{d}}}\vartheta \nonumber \\&\le n^{-2d}\Vert {\mathbf {e}}_\nu \Vert _{L^1(\mathbb {T}^d)}\le Cn^{-2d}. \end{aligned}$$

By means of (4.18) we get that

4.19 $$\begin{aligned} C\sum _{x,\,y\in \mathbb {T}^d_n}\sum _{w\in {{\mathrm{\mathbb {Z}}}}_n^d{\setminus } \{0\}}\left( 1-{\widehat{\phi _\kappa }}(w)\right) \frac{\exp (2\pi \iota (x-y)\cdot w)}{\Vert w\Vert ^4}F_{n,\,\nu }(x)\overline{F_{n,\,\nu }(y)}\le C\kappa . \end{aligned}$$

We are back to bounding the first term in (4.17).

$$\begin{aligned}&C\sum _{x,\,y\in \mathbb {T}^d_n}\sum _{w\in {{\mathrm{\mathbb {Z}}}}_n^d{\setminus } \{0\}}{\widehat{\phi _\kappa }}(w)\frac{\exp (2\pi \iota (x-y)\cdot w)}{\Vert w\Vert ^4}F_{n,\,\nu }(x)\overline{F_{n,\,\nu }(y)}\\&\quad =C\sum _{x,\,y\in \mathbb {T}^d_n}\sum _{w\in {{\mathrm{\mathbb {Z}}}}^d{\setminus }\{0\}}{\widehat{\phi _\kappa }}(w)\frac{\exp (2\pi \iota (x-y)\cdot w)}{\Vert w\Vert ^4}F_{n,\,\nu }(x)\overline{F_{n,\,\nu }(y)}\\&\qquad -C\sum _{x,\,y\in \mathbb {T}^d_n}\sum _{w\in {{\mathrm{\mathbb {Z}}}}^d:\,\Vert w\Vert _\infty >n}{\widehat{\phi _\kappa }}(w)\frac{\exp (2\pi \iota (x-y)\cdot w)}{\Vert w\Vert ^4}F_{n,\,\nu }(x)\overline{F_{n,\,\nu }(y)}. \end{aligned}$$

Using (4.9) we obtain a bound on the second term as

4.20 $$\begin{aligned}&\sum _{x,\,y\in \mathbb {T}^d_n}\sum _{w\in {{\mathrm{\mathbb {Z}}}}^d:\,\Vert w\Vert _\infty>n}{\widehat{\phi _\kappa }}(w)\frac{\exp (2\pi \iota (x-y)\cdot w)}{\Vert w\Vert ^4}F_{n,\,\nu }(x)\overline{F_{n,\,\nu }(y)}\nonumber \\&\quad \le C\sum _{x,\,y\in \mathbb {T}^d_n}\sum _{w\in {{\mathrm{\mathbb {Z}}}}^d:\,\Vert w\Vert _\infty>n}n^{-4}\left| {\widehat{\phi _\kappa }}(w)\right| \left| F_{n,\,\nu }(x)\overline{F_{n,\,\nu }(y)}\right| \nonumber \\&\quad \le C\sum _{w\in {{\mathrm{\mathbb {Z}}}}^d:\,\Vert w\Vert _\infty>n} \left| {\widehat{\phi _\kappa }}(w)\right| \left( \sum _{x\in \mathbb {T}_n^d} |F_{n,\,\nu }(x)|\right) ^2\nonumber \\&\quad \le C\sum _{w\in {{\mathrm{\mathbb {Z}}}}^d:\,\Vert w\Vert _\infty >n} \frac{\Vert {\mathbf {e}}_\nu \Vert _{L^1(\mathbb {T}^d)}^2}{(1+\Vert w\Vert )^{\delta }}\le C. \end{aligned}$$

Finally (4.9) tells us that

4.21 $$\begin{aligned}&\sum _{x,\,y\in \mathbb {T}^d_n}\sum _{w\in {{\mathrm{\mathbb {Z}}}}^d{\setminus }\{0\}}{\widehat{\phi _\kappa }}(w)\frac{\exp (2\pi \iota (x-y)\cdot w)}{\Vert w\Vert ^4}F_{n,\,\nu }(x)\overline{F_{n,\,\nu }(y)}\nonumber \\&\quad \le C\sum _{x,\,y\in \mathbb {T}_n^d} \sum _{w\in {{\mathrm{\mathbb {Z}}}}^d}\frac{1}{(1+\Vert w\Vert )^{\delta }}\left| F_{n,\,\nu }(x)\overline{F_{n,\,\nu }(y)}\right| \nonumber \\&\quad \le C\sum _{w\in {{\mathrm{\mathbb {Z}}}}^d} \frac{1}{\left( 1+\Vert w\Vert \right) ^{\delta }} \Vert {\mathbf {e}}_\nu \Vert _{L^1(\mathbb {T}^d)}^2\le C, \end{aligned}$$

where *C* possibly depends on $\kappa$ and $\delta$. Plugging in (4.15) the expressions (4.19), (4.20) and (4.21) we can draw the required conclusion. $\square$

This gives a proof of (P1) on page 7 and completes the proof of Theorem 1.

## Proof of Theorem 2

*Strategy of the proof* We will argue as in Theorem 1 and need thus to show both (P1) and (P2). While (P2) will follow almost in the same way as in the Gaussian case, (P1) will require a different approach. Firstly, we will need to remove constants in defining $e_n$ so that we will end up working with a field depending only on linear combinations of $(\sigma (x))_{x\in {{\mathrm{\mathbb {Z}}}}_n^d}$. Secondly, we will show in Sect. 5.1 that, for $\sigma$ bounded a. s., the convergence to the bilaplacian field is ensured via the moment method. Lastly, we will truncate the weights $\sigma$ at a level $\mathcal R>0$ and show that the truncated field approximates the original one.

*Reduction to a bounded field* We first recall some facts from Levine et al. [17]. Note that odometer $e_n$ satisfies

$$\begin{aligned} {\left\{ \begin{array}{ll} \Delta _g e_n(x)= 1-s(x), \\ \min _{z\in {{\mathrm{\mathbb {Z}}}}_n^d} e_n(z)=0. \end{array}\right. } \end{aligned}$$

Also if one defines

5.1 $$\begin{aligned} v_n(y)=\frac{1}{2d} \sum _{x\in {{\mathrm{\mathbb {Z}}}}_n^d} g(x,y) (s(x)-1), \end{aligned}$$

then $\Delta _g(e_n-v_n)(z)=0$. Since any harmonic function on a finite connected graph is constant, it follows from the proof of Proposition 1.3 of Levine et al. [17] that the odometer has the following representation also in the case where the weights are non-Gaussian:

5.2 $$\begin{aligned} e_n(x)= v_n(x)-\min _{z\in {{\mathrm{\mathbb {Z}}}}_n^d}v_n(z). \end{aligned}$$

Let us define the following functional: for any function $h_n: {{\mathrm{\mathbb {Z}}}}_n^d\rightarrow {{\mathrm{\mathbb {R}}}}$ set

$$\begin{aligned} \Xi _{h_n}(x):=4\pi ^2\sum _{z\in \mathbb {T}^d_n}n^{\frac{d-4}{2}}h_n({nz}){{\mathrm{\mathbb {1}}}}_{B\left( z,\,\frac{1}{2n}\right) }(x),\quad x\in \mathbb {T}^d. \end{aligned}$$

Note that for $u\in C^\infty (\mathbb {T}^d)$ such that $\int _{\mathbb {T}^d} u(x){{\mathrm{d}}}x=0$ it follows immediately that

$$\begin{aligned} \left\langle \Xi _{e_n}, u\right\rangle = \left\langle \Xi _{v_n}, u\right\rangle . \end{aligned}$$

Observe that

$$\begin{aligned} s(x)-1= \sigma (x)-\frac{1}{n^d} \sum _{y\in {{\mathrm{\mathbb {Z}}}}_n^d} \sigma (y) \end{aligned}$$

and hence we have from (5.1)

$$\begin{aligned} v_n(y)=\frac{1}{2d}\sum _{x\in {{\mathrm{\mathbb {Z}}}}_n^d} g(x,y)\sigma (x)-\frac{1}{2d n^{d}} \sum _{x\in {{\mathrm{\mathbb {Z}}}}_n^d} g(x,y) \sum _{z\in {{\mathrm{\mathbb {Z}}}}_n^d} \sigma (z). \end{aligned}$$

By (3.2) it follows that $(2d)^{-1} \sum _{x\in {{\mathrm{\mathbb {Z}}}}_n^d} g(x,y)= (2d)^{-1}n^{-d}\sum _{w\in {{\mathrm{\mathbb {Z}}}}_n^d} \mathsf {E}_y[\tau _w]$ which is independent of *y*. We can then say that

$$\begin{aligned} v_n(y)= \frac{1}{2d}\sum _{x\in {{\mathrm{\mathbb {Z}}}}_n^d} g(x,y)\sigma (x)- Cn^{-d}\sum _{z\in {{\mathrm{\mathbb {Z}}}}_n^d} \sigma (z). \end{aligned}$$

If we call

$$\begin{aligned} w_n(y):=\left( 2d\right) ^{-1}\sum _{x\in {{\mathrm{\mathbb {Z}}}}_n^d} g(x,y)\sigma (x), \end{aligned}$$

by the mean-zero property of the test functions it follows that $\left\langle \Xi _{v_n}, u\right\rangle = \left\langle \Xi _{w_n}, u \right\rangle .$ Therefore we shall reduce ourselves to study the convergence of the field $\Xi _{w_n}.$ To determine its limit, we will first prove that all moments of $\Xi _{w_n}$ converge to those of $\Xi$; via characteristic functions, we will show that the limit is uniquely determined by moments.

### Scaling limit with bounded weights

The goal of this Subsection is to determine the scaling limit for bounded weights, namely to prove

#### Theorem 11

(Scaling limit for bounded weights) Assume $(\sigma (x))_{x\in {{\mathrm{\mathbb {Z}}}}_n^d}$ is a collection of i.i.d. variables with $\mathsf {E}\left[ \sigma \right] =0$ and $\mathsf {E}\left[ \sigma ^2\right] =1$. Moreover assume there exists $K<+\infty$ such that $|\sigma |\le K$ almost surely. Let $d\ge 1$ and $e_n(\cdot )$ be the corresponding odometer. Then if we define the formal field $\Xi _n$ as in (1.3) for such i.i.d. weights, then it converges in law as $n\rightarrow +\infty$ to the bilaplacian field $\Xi$ on $\mathbb {T}^d$. The convergence holds in the same fashion of Theorem 1.

Before showing this result, we must prove an auxiliary Lemma. It gives us a uniform estimate in *n* on the Fourier series of the mean of *u* in a small ball.

#### Lemma 12

Fix $u\in C^\infty (\mathbb {T}^d)$ with zero average. If we define

$$\begin{aligned} T_n:\mathbb {T}^d&\rightarrow {{\mathrm{\mathbb {R}}}}\\ z&\mapsto \int _{B(z,\,\frac{1}{2n})}u(y){{\mathrm{d}}}y \end{aligned}$$

and ${\mathcal {T}}_n:{{\mathrm{\mathbb {Z}}}}_n^d\rightarrow {{\mathrm{\mathbb {R}}}}$ is defined as $\mathcal T_n(z):=T_n\left( \frac{z}{n}\right) ,$ then for *n* large enough we can find a constant $\mathcal M:=\mathcal M(d,\,u)<+\infty$ such that

$$\begin{aligned} n^{d}\sum _{z\in {{\mathrm{\mathbb {Z}}}}_n^d} \left| \widehat{ {\mathcal {T}}_n}(z)\right| \le \mathcal M. \end{aligned}$$

#### Proof

For $z\in {{\mathrm{\mathbb {Z}}}}_n^d$ we can write

5.3 $$\begin{aligned} \widehat{{\mathcal {T}}_n}(z)&= \left\langle {\mathcal {T}}_n, \,\psi _z\right\rangle = \frac{1}{n^d} \sum _{y\in {{\mathrm{\mathbb {Z}}}}_n^d} {\mathcal {T}}_n(y) \psi _{-z}(y)\nonumber \\&= \frac{1}{n^d} \sum _{y\in {{\mathrm{\mathbb {Z}}}}_n^d} T_n\left( \frac{y}{n}\right) \exp \left( -2\pi \iota z\cdot \frac{y}{n}\right) =\frac{1}{n^d} \sum _{y\in \mathbb {T}_n^d} T_n(y) \exp (-2\pi \iota z\cdot y).\nonumber \\ \end{aligned}$$

Since $u\in C^\infty (\mathbb {T}^d)$, one can take derive under the integral sign and get that $T_n\in C^\infty (\mathbb {T}^d)$, so $\sum _{z\in {{\mathrm{\mathbb {Z}}}}^d} \left| \widehat{T_n}(z)\right| <+\infty$. Hence by the Fourier inversion theorem we have the following inversion formula to be valid for every $y\in \mathbb {T}^d$:

$$\begin{aligned} T_n(y)=\sum _{w\in {{\mathrm{\mathbb {Z}}}}^d} \widehat{T_n}( w) \exp \left( 2\pi \iota y\cdot w\right) . \end{aligned}$$

First we split the sum above according to the norm of *w* and plug it in (5.3). Namely we get

5.4 $$\begin{aligned} \widehat{{\mathcal {T}}_n}(z)&= \frac{1}{n^d} \sum _{y\in \mathbb {T}_n^d} T_n(y) \exp (-2\pi \iota z\cdot y)\nonumber \\&= \frac{1}{n^d} \sum _{y\in \mathbb {T}_n^d} \sum _{ w\in {{\mathrm{\mathbb {Z}}}}_n^d} \widehat{T_n}(w) \exp (2\pi \iota w\cdot y)\exp (-2\pi \iota z\cdot y)\nonumber \\&\quad +\frac{1}{n^d} \sum _{y\in \mathbb {T}_n^d} \sum _{ w\in {{\mathrm{\mathbb {Z}}}}^d:\,\Vert w\Vert _\infty >n} \widehat{T_n}(w) \exp (2\pi \iota w\cdot y)\exp (-2\pi \iota z\cdot y). \end{aligned}$$

Let us look at the first summation: using the orthogonality of the characters of $L^2({{\mathrm{\mathbb {Z}}}}^d_n)$ we can write

$$\begin{aligned}&\frac{1}{n^d} \sum _{y\in \mathbb {T}_n^d} \sum _{ w\in {{\mathrm{\mathbb {Z}}}}_n^d} \widehat{T_n}(w) \exp (2\pi \iota w\cdot y)\exp (-2\pi \iota z\cdot y)\\&\quad = \frac{1}{n^d} \sum _{ w\in {{\mathrm{\mathbb {Z}}}}_n^d} \widehat{T_n}(w) \sum _{y\in {{\mathrm{\mathbb {Z}}}}_n^d}\exp \left( 2\pi \iota w\cdot \frac{y}{n}\right) \exp \left( -2\pi \iota z\cdot \frac{y}{n}\right) \\&\quad = \frac{1}{n^d} \sum _{w\in {{\mathrm{\mathbb {Z}}}}_n^d} \widehat{T}_n(w) n^d{{\mathrm{\mathbb {1}}}}_{w=z}= \widehat{T_n}(z). \end{aligned}$$

Noting that

$$\begin{aligned} \widehat{{\mathcal {T}}_n}(0) =\frac{1}{n^d} \sum _{y\in \mathbb {T}_n^d} T_n(y)=\frac{1}{n^d}\sum _{y\in \mathbb {T}_n^d} \int _{B\left( y, \frac{1}{2n}\right) }u(x){{\mathrm{d}}}x=\frac{1}{n^d}\int _{\mathbb {T}^d} u(x){{\mathrm{d}}}x=0, \end{aligned}$$

this means we need to show that $\sum _{z\in {{\mathrm{\mathbb {Z}}}}_n^d{\setminus } \{0\}} \left| \widehat{T_n}(z)\right| \le C(d)n^{-d}$. We follow the proof of Stein and Weiss [26, Corollary 1.9, Chapter VII]. For a multi-index $\alpha =(\alpha _1,\,\ldots ,\,\alpha _d)\in {{\mathrm{\mathbb {N}}}}^d$ and a point $x=(x_1,\,\ldots ,\,x_d)\in {{\mathrm{\mathbb {R}}}}^d$ we set

$$\begin{aligned} x^\alpha :=\prod _{j=1}^d x_j^{\alpha _j} \end{aligned}$$

and adopt the convention $0^0=1$. We choose now a smoothness parameter $k_0>d$. For any $\alpha$ with $|\alpha |:=\alpha _1+\cdots +\alpha _d\le k_0$ we can find a constant $c=c(k_0,\,d)$ such that

$$\begin{aligned} \sum _{\alpha :\,|\alpha |=k_0}4\pi ^2z^{2\alpha }\ge c\Vert z\Vert ^{2k_0}. \end{aligned}$$

Note that

$$\begin{aligned}&\sum _{z\in {{\mathrm{\mathbb {Z}}}}_n^d{\setminus } \{0\}} \left| \widehat{T_n} (z)\right| \le \sum _{z\in {{\mathrm{\mathbb {Z}}}}_n^d{\setminus } \{0\}} \left| \widehat{T_n} (z)\right| \left( \sum _{\alpha :\,|\alpha |=k_0} 4\pi ^2 z^{2\alpha }\right) ^{\frac{1}{2}}\Vert z\Vert ^{-k_0}c^{-\frac{1}{2}}\\&\qquad \le \left( \sum _{z\in {{\mathrm{\mathbb {Z}}}}_n^d{\setminus } \{0\}} \left| \widehat{T_n} (z)\right| ^2 \sum _{\alpha :\,|\alpha |=k_0} 4\pi ^2 z^{2\alpha }\right) ^{\frac{1}{2}} \left( \sum _{z\in {{\mathrm{\mathbb {Z}}}}_n^d{\setminus } \{0\}} \Vert z\Vert ^{-2k_0}\right) ^{\frac{1}{2}}c^{-\frac{1}{2}}. \end{aligned}$$

Here we have used the Cauchy-Schwarz inequality in the last step. Now since $\sum _{z\in {{\mathrm{\mathbb {Z}}}}_n^d{\setminus } \{0\}} \Vert z\Vert ^{-2k_0}<+\infty$ we can compute a constant *C* such that

5.5 $$\begin{aligned}&\sum _{z\in {{\mathrm{\mathbb {Z}}}}_n^d{\setminus } \{0\}} \left| \widehat{T_n} (z)\right| \le C \left( \sum _{z\in {{\mathrm{\mathbb {Z}}}}_n^d{\setminus } \{0\}} \left| \widehat{T_n} (z)\right| ^2 \sum _{\alpha :\,|\alpha |=k_0} 4\pi ^2 z^{2\alpha }\right) ^{\frac{1}{2}}\nonumber \\&\qquad \le C\left( \sum _{\alpha :\,|\alpha |=k_0} \sum _{z\in {{\mathrm{\mathbb {Z}}}}^d} \left| \widehat{T_n} (z)\right| ^2 4\pi ^2 z^{2\alpha }\right) ^{\frac{1}{2}}. \end{aligned}$$

Let us call $D^\alpha$ the derivative with respect to $\alpha$. Using the rule of derivation of Fourier transforms [26, Chapter I, Theorem 1.8] and Parseval we have that

$$\begin{aligned} \sum _{z\in {{\mathrm{\mathbb {Z}}}}^d} \left| \widehat{T_n} (z)\right| ^2 4\pi ^2 z^{2\alpha }= \int _{\mathbb {T}^d} \left| D^\alpha T_n(x)\right| ^2{{\mathrm{d}}}x. \end{aligned}$$

By the smoothness of *u* we deduce that

5.6 $$\begin{aligned} |D^\alpha T_n(x)| \le \Vert D^\alpha u\Vert _{L^\infty (\mathbb {T}^d)} \int _{B(0,\frac{1}{2n})} {{\mathrm{d}}}w= \Vert D^\alpha u\Vert _{L^\infty (\mathbb {T}^d)} (2n)^{-d}. \end{aligned}$$

Plugging this estimate in (5.5) we get that

$$\begin{aligned} \sum _{z\in {{\mathrm{\mathbb {Z}}}}_n^d{\setminus }\{0\}} \left| \widehat{T_n} (z)\right| ^2 \le C n^{-{d}} \left( \sum _{\alpha :\,|\alpha |=k_0}\Vert D^\alpha u\Vert _{L^\infty (\mathbb {T}^d)}^2 \right) ^{\frac{1}{2}}. \end{aligned}$$

This finally gives that

$$\begin{aligned} \sum _{z\in {{\mathrm{\mathbb {Z}}}}_n^d{\setminus } \{0\}} \left| \widehat{T_n} (z)\right| \le C(k_0,\,d,\,u) n^{-d}. \end{aligned}$$

For the second summand of (5.4) observe that

$$\begin{aligned} \int _{\mathbb {T}^d}{D^\alpha T_n}(w){{\mathrm{e}}}^{-2\pi \iota z\cdot w}{{\mathrm{d}}}w=(2\pi \iota z)^\alpha \widehat{T_n}(z),\quad \alpha \in {{\mathrm{\mathbb {N}}}}^d. \end{aligned}$$

The parameter $\alpha$ will be chosen later so that the second summand is of lower order than the first. By (5.4) and (5.6)

$$\begin{aligned} \left| \widehat{T_n}(z)\right| \le \frac{2^{-d-1}\Vert D^\alpha u\Vert _{L^\infty (\mathbb {T}^d)}}{ \pi n^d\left| z^\alpha \right| }. \end{aligned}$$

We use this estimate to get

$$\begin{aligned}&\frac{1}{n^d} \sum _{y\in \mathbb {T}_n^d} \sum _{\Vert w\Vert _\infty> n} \widehat{T_n}(w) \exp (2\pi \iota w\cdot y)\exp (-2\pi \iota z\cdot y)\le \sum _{\Vert w\Vert _\infty > n} \left| \widehat{T_n}(w)\right| \\&\quad \le \frac{C(u,\,d,\,\alpha )}{ n^d}\sum _{\ell =n}^{+\infty }\frac{\ell ^{d-1}}{\ell ^{|\alpha |}}\le C(u,\,d,\,\alpha )n^{-|\alpha |}\left( 1+\mathrm {O}\left( n^{-1}\right) \right) . \end{aligned}$$

Thus choosing $\alpha$ with $|\alpha |>d$ we find a constant $\mathcal M=\mathcal M(d,\,u)$ such that

$$\begin{aligned} \sum _{z\in {{\mathrm{\mathbb {Z}}}}_n^d} \left| \widehat{{\mathcal {T}}_n}(z)\right| \le \mathcal M n^{-d} \end{aligned}$$

as we wanted to show. $\square$

We can now start with the moment method, and we being with moment convergence.

*Moment convergence * We now show that all moments converge to those of the required limiting distribution. This is explained in the following Proposition.

#### Proposition 13

Assume $\mathsf {E}\left[ \sigma \right] =0$, $\mathsf {E}\left[ \sigma ^2\right] =1$ and that there exists $K<+\infty$ such that $|\sigma |\le K$ almost surely. Then for all $m\ge 1$ and all $u\in C^\infty (\mathbb {T}^d)$ with zero average, the following limits hold:

5.7 $$\begin{aligned} \lim _{n\rightarrow +\infty }\mathsf {E}\left[ \left\langle \Xi _{w_n}, u\right\rangle ^{m}\right] ={\left\{ \begin{array}{ll} (2m-1)!! \Vert u\Vert _{-1}^{m},&{}m\in 2{{\mathrm{\mathbb {N}}}}\\ 0,&{}m\in 2{{\mathrm{\mathbb {N}}}}+1. \end{array}\right. } \end{aligned}$$

#### Proof

We will first show that the $m=2$ case satisfies the claim.

*Case* $m=2$ We have the equality

$$\begin{aligned} \mathsf {E}\left[ w_n(y)w_n(y')\right]&=(2d)^{-2} \sum _{x\in {{\mathrm{\mathbb {Z}}}}_n^d} g(x,y)\sum _{x'\in {{\mathrm{\mathbb {Z}}}}_n^d} g(x',y') \mathsf {E}[\sigma (x)\sigma (x')]. \end{aligned}$$

The independence of the weights gives

$$\begin{aligned} \mathsf {E}\left[ \left\langle \Xi _{w_n}, u\right\rangle ^2\right] =16\pi ^4 \frac{n^{d-4}}{4 d^2} \sum _{x\in {{\mathrm{\mathbb {Z}}}}_n^d}\left( \sum _{z\in \mathbb {T}_n^d}g(x,\,nz)T_n(z)\right) ^2. \end{aligned}$$

With the same argument of the proof of Proposition 4 one has

5.8 $$\begin{aligned} (2d)^{-2} \sum _{x\in {{\mathrm{\mathbb {Z}}}}_n^d} g(x,y)g(x,y')= n^d L^2+ H(y,y') \end{aligned}$$

so that, using that test functions have zero average,

$$\begin{aligned} \mathsf {E}\left[ \left\langle \Xi _{w_n}, u\right\rangle ^2\right]&=\,16\pi ^4 \frac{n^{d-4}}{4 d^2} \sum _{x\in {{\mathrm{\mathbb {Z}}}}_n^d}\left( \sum _{z\in \mathbb {T}_n^d}g(x,\,nz)T_n(z)\right) ^2\\&=16\pi ^4 {n^{d-4}} \sum _{z,\,z'\in \mathbb {T}_n^d} H(nz, nz')T_n(z)T_n(z')\\&=16\pi ^4 {n^{d-4}} \sum _{z,\,z'\in \mathbb {T}_n^d} H(nz, nz') \int _{B(z,\,\frac{1}{2n})} u(x){{\mathrm{d}}}x\int _{B(z',\,\frac{1}{2n})} u(x'){{\mathrm{d}}}x'. \end{aligned}$$

Now we break the above sum into the following 3 sums (recall $K_n(u)$ from (4.2)):

$$\begin{aligned} \mathsf {E}\left[ \left\langle \Xi _{w_n}, u\right\rangle ^2\right]&= 16\pi ^4 n^{d-4} \sum _{z,\,z'\in \mathbb {T}_n^d}n^{-2d} H(nz, nz') u(z) u(z')\\&\quad +16\pi ^4 n^{d-4} \sum _{z,\,z'\in \mathbb {T}_n^d}n^{-2d} H(nz, nz') K_n(z) K_n(z')\\&\quad +32\pi ^4 n^{d-4} \sum _{z,\,z'\in \mathbb {T}_n^d}n^{-2d} H(nz, nz') K_n(z) u(z'). \end{aligned}$$

A combination of Propositions 5 and 6 with the Cauchy-Schwarz inequality shows that the first term converges to $\Vert u\Vert ^2_{-1}$ in the limit $n\rightarrow +\infty$ and the other two go to zero.

Having concluded the case $m=2$, we would like to see what the higher moments look like. Let us take for example $m=3$, in which case

$$\begin{aligned}&\mathsf {E}\left[ \left\langle \Xi _{w_n}, u\right\rangle ^3\right] \\&\quad = \left( \frac{4\pi ^2 n^{\frac{d-4}{2}}}{2d}\right) ^3 \sum _{z_1, \,z_2, \,z_3\in \mathbb {T}_n^d} \mathsf {E}\left[ w(nz_1)w(nz_2)w(nz_3)\right] T_n(z_1) T_n(z_2)T_n(z_3)\\&\quad =\left( \frac{2\pi ^2 n^{\frac{d-4}{2}}}{d}\right) ^3 \sum _{z_1, \,z_2, \,z_3\in \mathbb {T}_n^d} \sum _{x_1,\,x_2,\,x_3\in {{\mathrm{\mathbb {Z}}}}_n^d}\mathsf {E}\left[ \prod _{j=1}^3\sigma (x_j) \right] \prod _{j=1}^3g(x_j,\,nz_j)T_n(z_j)\\&\quad = \left( \frac{2\pi ^2 n^{\frac{d-4}{2}}}{d}\right) ^3 \sum _{z_1, \,z_2, \,z_3\in \mathbb {T}_n^d} \sum _{x \in {{\mathrm{\mathbb {Z}}}}_n^d}\mathsf {E}\left[ \sigma ^3(x) \right] \prod _{j=1}^3g(x,\,nz_j)T_n(z_j)\\&\quad = \left( \frac{2\pi ^2 n^{\frac{d-4}{2}}}{d}\right) ^3 \mathsf {E}\left[ \sigma ^3 \right] \sum _{x \in {{\mathrm{\mathbb {Z}}}}_n^d} \left[ \sum _{z \in \mathbb {T}_n^d} g(x,\,nz)T_n(z)\right] ^3. \end{aligned}$$

More generally, let us call ${\mathscr {P}}(n)$ the set of partitions of $\{1,\,\ldots ,\,n\}$ and as ${\mathscr {P}}_2(n)\subset {\mathscr {P}}(n)$ the set of pair partitions. We denote as $\Pi$ a generic block of a partition *P* and as $|\Pi |$ its cardinality (for example, $\Pi =\{1,\,2,\,3\}$ is a block of cardinality 3 of $P=\{\{1,\,2,\,3\},\,\{4\}\}\in {\mathscr {P}}(4)$). Observe that

5.9 $$\begin{aligned}&\mathsf {E}\left[ \left\langle \Xi _{w_n}, u\right\rangle ^{m}\right] =\left( \frac{2\pi ^2 n^{\frac{d-4}{2}}}{d}\right) ^m\sum _{z_1,\,\ldots ,\,z_m\in \mathbb {T}_n^d}\mathsf {E}\left[ \prod _{j=1}^m w_n(nz_j)\right] \prod _{j=1}^m T_n(z_j)\nonumber \\&\quad =\left( \frac{2\pi ^2 n^{\frac{d-4}{2}}}{d}\right) ^m\sum _{P\in {\mathscr {P}}(m)}\prod _{\Pi \in P}\mathsf {E}\left[ \sigma ^{|\Pi |}\right] \sum _{x \in {{\mathrm{\mathbb {Z}}}}_n^d }\left( \sum _{ \begin{array}{c} z_j \in \mathbb {T}^d_n:\,j \in \Pi \end{array}} \,\prod _{j\in \Pi } g(x,\, nz_j)T_n(z_j)\right) \nonumber \\&\quad =\sum _{P\in {\mathscr {P}}(m)}\prod _{\Pi \in P}\left( \frac{2\pi ^2 n^{\frac{d-4}{2}}}{d}\right) ^{|\Pi |}\mathsf {E}\left[ \sigma ^{|\Pi |}\right] \sum _{x \in {{\mathrm{\mathbb {Z}}}}_n^d } \left( \sum _{z \in \mathbb {T}^d_n} g(x,\,nz)T_n(z) \right) ^{|\Pi |}. \end{aligned}$$

For a fixed *P*, let us consider in the product over $\Pi \in P$ any term corresponding to a block $\Pi$ with $|\Pi |=1$: this will give no contribution because $\sigma$ is centered. Consider instead $\Pi \in P$ with $\ell :=|\Pi |>2.$ We see that

$$\begin{aligned}&\left( \frac{2\pi ^2 n^{\frac{d-4}{2}}}{d}\right) ^\ell \mathsf {E}\left[ \sigma ^\ell \right] \sum _{x\in {{\mathrm{\mathbb {Z}}}}_n^d} \left( \sum _{z \in \mathbb {T}^d_n} g(x,\,nz)T_n(z) \right) ^{l} \nonumber \\&\quad =\left( \frac{2\pi ^2 n^{\frac{d-4}{2}}}{d}\right) ^\ell \mathsf {E}\left[ \sigma ^\ell \right] \sum _{x\in {{\mathrm{\mathbb {Z}}}}_n^d}\left( \sum _{z\in {{\mathrm{\mathbb {Z}}}}_n^d}g(x,\,z){{\mathcal {T}}_n}(z)\right) ^\ell . \end{aligned}$$

Applying Parseval the above expression equals

5.10 $$\begin{aligned}&\left( \frac{2\pi ^2 n^{\frac{d-4}{2}}}{d}\right) ^\ell \mathsf {E}\left[ \sigma ^\ell \right] \sum _{x\in {{\mathrm{\mathbb {Z}}}}_n^d}\left( n^{d}\sum _{z\in {{\mathrm{\mathbb {Z}}}}_n^d}\widehat{g_x}(z) \widehat{{\mathcal {T}}_n}(z)\right) ^\ell \nonumber \\&\quad {\mathop {=}\limits ^{(2.2)}}\left( {4\pi ^2 n^{\frac{d-4}{2}}}\right) ^\ell \mathsf {E}\left[ \sigma ^\ell \right] \sum _{x\in {{\mathrm{\mathbb {Z}}}}_n^d}\left( \sum _{z\in {{\mathrm{\mathbb {Z}}}}_n^d{\setminus }\{0\}}\frac{\psi _{-z}(x)}{-\lambda _z} \widehat{{\mathcal {T}}_n}(z)\right) ^\ell . \end{aligned}$$

Here we have used that $\widehat{{\mathcal {T}}_n}(0)=0.$ Thanks to the fact that $-\lambda _z\ge Cn^{-2}$ uniformly over $z\in {{\mathrm{\mathbb {Z}}}}_n^d{\setminus } \{0\}$ (see (4.5)) we obtain

5.11 $$\begin{aligned}&\left( \frac{2\pi ^2 n^{\frac{d-4}{2}}}{d}\right) ^\ell \mathsf {E}\left[ \sigma ^\ell \right] \sum _{x\in {{\mathrm{\mathbb {Z}}}}_n^d}\left( \sum _{z \in \mathbb {T}^d_n} g(x,\,nz)T_n(z) \right) ^{l}\nonumber \\&\quad \le C\mathsf {E}\left[ \sigma ^\ell \right] n^{\frac{\ell d}{2}+d} \left( \sum _{z\in {{\mathrm{\mathbb {Z}}}}_n^d{\setminus }\{0\}} \left| \widehat{\mathcal T_n}(z)\right| \right) ^\ell . \end{aligned}$$

Since $\sigma$ is almost surely bounded, by Lemma 12 we can conclude that each term in (5.9) corresponding to a block of cardinality $\ell >2$ has order at most $n^{\frac{ \ell d}{2}-(\ell -1)d}=\mathrm {o}\left( 1\right)$. Hence in (5.9) only pair partitions of *m* will give a contribution of order unity to the sum. Since, for $m:=2m'+1$, there are no pair partitions, $\mathsf {E}\left[ \left\langle \Xi _{w_n}, u\right\rangle ^{2m'+1}\right]$ will converge to zero. Otherwise, for $m:=2m'$ we can rewrite

$$\begin{aligned} \mathsf {E}\left[ \left\langle \Xi _{w_n}, u\right\rangle ^{2m'}\right]&=\sum _{P\in \mathscr {P}_2(2m')}\left( \frac{4\pi ^4 n^{{d-4}}}{d^2}\sum _{x\in {{\mathrm{\mathbb {Z}}}}_n^d }\left( \sum _{z\in {{\mathrm{\mathbb {Z}}}}_n^d}g(x,\,z){\mathcal {T}}_n(z)\right) ^2\right) ^{m'}+\mathrm {o}\left( 1\right) . \end{aligned}$$

Since $\left| {\mathscr {P}}_2(m)\right| =(2m-1)!!$ and the term in the bracket above converges to $\Vert u\Vert _{-1}^{2}$ we can conclude the proof of Proposition 13. $\square$

*Tightness* The proof of tightness is, not suprisingly, a re-run of that in the Gaussian case. In fact tightness depends on the covariance structure of the field we are examining; since both the Gaussian functional $\Xi _n$ and $w_n$ share the same covariance, we can recover mostly of the results already calculated. First we notice that

$$\begin{aligned} ||{\Xi _{w_n}}||_{L^2(\mathbb {T}^d)}^2=\frac{16\pi ^4}{(2d)^2} n^{d-4}\sum _{x,\,y\in {{\mathrm{\mathbb {Z}}}}_n^d}g(x,\,y)\sigma (x)\sum _{x',\,y'\in {{\mathrm{\mathbb {Z}}}}_n^d}g(x',\,y')\sigma (x') \end{aligned}$$

is finite with probability one, since $\sigma$ is bounded. One can then go along the lines of the proof of (P1) in Sect. 4.2 and get to (4.14) which will become, in our new setting,

$$\begin{aligned}&\frac{16\pi ^4}{(2d)^2}\sum _{\nu \in {{\mathrm{\mathbb {Z}}}}^d{\setminus }\{0\}}\sum _{x,\,y\in \mathbb {T}^d_n}\Vert \nu \Vert ^{-2\epsilon }n^{d-4}\mathsf {E}\left[ w_n(nx)w_n(ny)\right] \int _{B(x,\,\frac{1}{2n})}{\mathbf {e}}_{\nu }(\vartheta ){{\mathrm{d}}}\vartheta \int _{B(y,\,\frac{1}{2n})}\overline{{\mathbf {e}}_{\nu }(\vartheta )}{{\mathrm{d}}}\vartheta \\&\quad {\mathop {=}\limits ^{(5.8)}}{16\pi ^4}\sum _{\nu \in {{\mathrm{\mathbb {Z}}}}^d{\setminus }\{0\}}\sum _{x,\,y\in \mathbb {T}^d_n}\Vert \nu \Vert ^{-2\epsilon }n^{d-4}\left( n^d L^2+H(nx,\,ny)\right) \nonumber \\&\qquad \times \int _{B(x,\,\frac{1}{2n})}{\mathbf {e}}_{\nu }(\vartheta ){{\mathrm{d}}}\vartheta \int _{B(y,\,\frac{1}{2n})}\overline{{\mathbf {e}}_{\nu }(\vartheta )}{{\mathrm{d}}}\vartheta . \end{aligned}$$

Since $\int _{\mathbb {T}^d}{\mathbf {e}}_{\nu }(\vartheta ){{\mathrm{d}}}\vartheta =0$, the previous expression reduces to

$$\begin{aligned} {16\pi ^4}\sum _{\nu \in {{\mathrm{\mathbb {Z}}}}^d{\setminus }\{0\}}\sum _{x,\,y\in \mathbb {T}^d_n}\Vert \nu \Vert ^{-2\epsilon }n^{d-4}H(nx,\,ny)\int _{B(x,\,\frac{1}{2n})}{\mathbf {e}}_{\nu }(\vartheta ){{\mathrm{d}}}\vartheta \int _{B(y,\,\frac{1}{2n})}\overline{{\mathbf {e}}_{\nu }(\vartheta )}{{\mathrm{d}}}\vartheta . \end{aligned}$$

From this point onwards, the computations of the proof of (P1) can be repeated in a one-to-one fashion.

### Truncation method

At the moment we are able to determine the scaling limit when the weights are bounded almost surely. To lift this condition to zero mean and finite variance only, we begin by defining a truncated field and show it will determine the scaling limit of the global field. Fix an arbitrarily large (but finite) constant $\mathcal R>0$. Set

$$\begin{aligned} w_n^{<\mathcal R}(x)&:=\frac{1}{2d}\sum _{y\in {{\mathrm{\mathbb {Z}}}}_n^d}g(x,\,y)\sigma (y){{\mathrm{\mathbb {1}}}}_{\{|\sigma (y)|< \mathcal R\}},\\ w_n^{\ge \mathcal R}(x)&:=\frac{1}{2d}\sum _{y\in {{\mathrm{\mathbb {Z}}}}_n^d}g(x,\,y)\sigma (y){{\mathrm{\mathbb {1}}}}_{\{|\sigma (y)|\ge \mathcal R\}}. \end{aligned}$$

Clearly $w_n(\cdot )=w_n^{<\mathcal R}(\cdot )+w_n^{\ge \mathcal R}(\cdot )$. To prove our result, we will use

#### Theorem 14

(Billingsley [3, Theorem 4.2]) Let *S* be a metric space with metric $\rho$. Suppose that $(X_{n,\,u},\,X_n)$ are elements of $S\times S.$ If

$$\begin{aligned} \lim _{u\rightarrow +\infty } \limsup _{n\rightarrow +\infty } \mathsf {P}\left( \rho (X_{n,\,u},\,X_n)\ge \tau \right) =0 \end{aligned}$$

for all $\tau >0$, and $X_{n,\,u}\Rightarrow _{n}Z_u\Rightarrow _u X$, where $``\Rightarrow _x''$ indicates convergence in law as $x\rightarrow +\infty$, then $X_n\Rightarrow _n X$.

Following this Theorem, we need to show two steps:

1. $$\lim _{\mathcal R\rightarrow +\infty } \limsup _{n\rightarrow +\infty } \mathsf {P}\left( \left\| \Xi _{w_n}-\Xi _{w_n^{<\mathcal R}}\right\| _{\mathcal H_{-\epsilon }}\ge \tau \right) =0$$ for all $$\tau >0$$.
2. For a constant $v_\mathcal R>0$, we have $\Xi _{w_n^{<\mathcal R}}\Rightarrow _n \sqrt{v_\mathcal R}\, \Xi \Rightarrow _{\mathcal R}\Xi$ in the topology of ${\mathcal {H}}_{-\epsilon }$.

As a consequence we will obtain that $\Xi _{w_n}$ converges to $\Xi$ in law in the topology of ${\mathcal {H}}_{-\epsilon }.$

#### Proof of (S1)

We notice that

$$\begin{aligned} \left\| \Xi _{w_n}-\Xi _{w_n^{<\mathcal R}}\right\| _{\mathcal H_{-\epsilon }}=\left\| \Xi _{w_n^{\ge \mathcal R}}\right\| _{{\mathcal {H}}_{-\epsilon }} \end{aligned}$$

by definition, for every realization of $(\sigma (x))_{x\in {{\mathrm{\mathbb {Z}}}}_n^d}$. Since, for every $\tau >0$,

$$\begin{aligned} \mathsf {P}\left( \left\| \Xi _{w_n^{\ge \mathcal R}}\right\| _{\mathcal H_{-\epsilon }}\ge \tau \right) \le \frac{\mathsf {E}\left[ \left\| \Xi _{w_n^{\ge \mathcal R}}\right\| _{{\mathcal {H}}_{-\epsilon }}^2\right] }{\tau ^2} \end{aligned}$$

it will suffice to show that the numerator on the right-hand side goes to zero to show (S1). But

5.12 $$\begin{aligned}&\mathsf {E}\left[ \left\| \Xi _{w_n^{\ge \mathcal R}}\right\| _{\mathcal H_{-\epsilon }}^2\right] \nonumber \\&\quad =16\pi ^4\sum _{\nu \in {{\mathrm{\mathbb {Z}}}}^d{\setminus }\{0\}}\sum _{x,\,y\in \mathbb {T}^d_n}\Vert \nu \Vert ^{-4\epsilon }n^{d-4}\mathsf {E}\left[ w_n^{\ge \mathcal R}(xn) w_n^{\ge \mathcal R}(ny)\right] \nonumber \\&\qquad \times \int _{B(x,\,\frac{1}{2n})}{\mathbf {e}}_{\nu }(\vartheta ){{\mathrm{d}}}\vartheta \int _{B(y,\,\frac{1}{2n})}\overline{{\mathbf {e}}_{\nu }(\vartheta )}{{\mathrm{d}}}\vartheta \end{aligned}$$

Since the $\sigma$’s are i.i.d., we see that

5.13 $$\begin{aligned}&\mathsf {E}\left[ w_{n}^{\ge R}(xn)w_{n}^{\ge \mathcal R}(yn)\right] =\frac{1}{4d^{2}}\sum _{w\in \mathbb {Z}_n^{d}}g(nx,\,w)g(ny,\,w)\mathsf {E}\left[ \sigma (w)^{2}\mathbb {1}_{\left\{ |\sigma (w)|\ge \mathcal R\right\} }\right] \nonumber \\&\qquad +\frac{1}{4d^{2}}\sum _{w\ne v\in \mathbb {Z}_n^{d}}g(nx,\,w)g(ny,\,v)\mathsf {E}\left[ \sigma (w)\sigma (v)\mathbb {1}_{\left\{ |\sigma (w)|\ge \mathcal R\right\} }\mathbb {1}_{\left\{ |\sigma (v)|\ge \mathcal R\right\} }\right] \nonumber \\&\quad =\left( \mathsf {E}\left[ \sigma ^2\mathbb {1}_{\left\{ |\sigma |\ge \mathcal R\right\} }\right] -\mathsf {E}\left[ \sigma \mathbb {1}_{\left\{ |\sigma |\ge \mathcal R\right\} }\right] ^{2}\right) \frac{1}{4d^{2}}\sum _{w\in \mathbb {Z}_n^{d}}g(nx,\,w)g(ny,\,w)\nonumber \\&\qquad +\mathsf {E}\left[ \sigma \mathbb {1}_{\left\{ |\sigma |\ge \mathcal R\right\} }\right] ^2\frac{1}{4d^{2}}\sum _{w,\,v\in \mathbb {Z}^{d}_n}g(nx,\,w)g(ny,\,v). \end{aligned}$$

Pluging the last expression into (5.12) gives two terms. The first one is, using (5.8), equal to

$$\begin{aligned}&16\pi ^4\left( \mathsf {E}\left[ \sigma ^{2}\mathbb {1}_{\left\{ |\sigma |\ge \mathcal R\right\} }\right] -E\left[ \sigma \mathbb {1}_{\left\{ |\sigma |\ge \mathcal R\right\} }\right] ^{2}\right) \\&\quad \times \sum _{\nu \in {{\mathrm{\mathbb {Z}}}}^d{\setminus }\{0\}}\Vert \nu \Vert ^{-4\epsilon }n^{d-4}\sum _{x,\,y\in \mathbb {T}^d_n}H(nx,\,ny)F_{n,\,\nu }(x)\overline{F_{n,\,\nu }(y)} \end{aligned}$$

where $F_{n,\,\nu }(x)$ was defined as $\int _{B(x,\,\frac{1}{2n})}{\mathbf {e}}_{\nu }(\vartheta ){{\mathrm{d}}}\vartheta$. We have at hand (4.15), which we can use to upper-bound the previous expression by

$$\begin{aligned} C'16\pi ^4\left( \mathsf {E}\left[ \sigma (w)^{2}\mathbb {1}_{\left\{ |\sigma (w)|\ge \mathcal R\right\} }\right] -E\left[ \sigma (w)\mathbb {1}_{\left\{ |\sigma (w)|\ge \mathcal R\right\} }\right] ^{2}\right) \sum _{\nu \in {{\mathrm{\mathbb {Z}}}}^d{\setminus }\{0\}}\Vert \nu \Vert ^{-4\epsilon } \end{aligned}$$

for some $C'>0$. The sum over $\nu$ is finite as long as $\epsilon >{d}/{4}$, and

$$\begin{aligned} \mathsf {E}\left[ \sigma (w)^{2}\mathbb {1}_{\left\{ |\sigma (w)|\ge \mathcal R\right\} }\right] -E\left[ \sigma (w)\mathbb {1}_{\left\{ |\sigma (w)|\ge \mathcal R\right\} }\right] ^{2} \end{aligned}$$

is going to zero as $\mathcal R\rightarrow +\infty$ (note that $\sigma$ has finite variance). We will show that the second term obtained by inserting the second summand of (5.13) in (5.12) is zero to complete the proof of (S1). In fact we obtain

$$\begin{aligned}&\frac{4\pi ^4}{d^2}n^{d-4}\mathsf {E}\left[ \sigma \mathbb {1}_{\left\{ |\sigma |\ge \mathcal R\right\} }\right] ^{2}\sum _{\nu \in {{\mathrm{\mathbb {Z}}}}^d{\setminus }\{0\}}\Vert \nu \Vert ^{-4\epsilon }\\&\quad \times \sum _{x,\,y\in \mathbb {T}^d_n}\sum _{w,\,v\in \mathbb {Z}_n^{d}}g(nx,\,w)g(ny,\,v) F_{n,\,\nu }(x)\overline{F_{n,\,\nu }(y)}. \end{aligned}$$

We consider the second line in the previous expression to deduce that it equals

$$\begin{aligned}&\left| \sum _{x\in \mathbb {T}^d_n}\sum _{w\in \mathbb {Z}_n^{d}}g(nx,\,w)F_{n,\,\nu }(x)\right| ^2=n^{2d}\left| \sum _{w\in \mathbb {Z}_n^{d}}\sum _{x\in {{\mathrm{\mathbb {Z}}}}^d_n}\widehat{g_w}(x)\widehat{F_{n,\,\nu }}(x)\right| ^2\\&\quad {\mathop {=}\limits ^{(2.2)}}\left| -2d\sum _{w\in \mathbb {Z}_n^{d}}\sum _{x\in {{\mathrm{\mathbb {Z}}}}^d_n{\setminus }\{0\}}\frac{\psi _{-x}(w)}{\lambda _x}\widehat{F_{n,\,\nu }}(x)+\sum _{w\in {{\mathrm{\mathbb {Z}}}}_n^d}\widehat{g_w}(0)\widehat{F_{n,\,\nu }}(0)\right| ^2 \end{aligned}$$

where Parseval’s theorem was used in the first equality. Both the summands above are zero: the first because

$$\begin{aligned} \sum _{w\in {{\mathrm{\mathbb {Z}}}}_n^d}\psi _{-x}(w)=n^d\langle \psi _0,\,\psi _{-x}\rangle =0,\quad x\ne 0, \end{aligned}$$

the second because ${\mathbf {e}}_{\nu }$ has zero average and so

$$\begin{aligned} \widehat{F_{n,\,\nu }}(0)=n^{-d}\sum _{y\in {{\mathrm{\mathbb {Z}}}}_n^d}F_{n, \,\nu }(y)=0. \end{aligned}$$

#### Proof of (S2)

Our idea is to use the computations we did for the case in which $\sigma$ is bounded a. s. since we are imposing that $|\sigma |<\mathcal R$. However we have to pay attention to the fact that $\sigma {{\mathrm{\mathbb {1}}}}_{\{|\sigma |<\mathcal R\}}$ is not centered anymore, but has mean $m_\mathcal R:=\mathsf {E}[\sigma {{\mathrm{\mathbb {1}}}}_{\{|\sigma |<\mathcal R\}}]$, nor has variance 1, but $v_{\mathcal R}:=\mathsf {Var}[\sigma {{\mathrm{\mathbb {1}}}}_{\{|\sigma |<\mathcal R\}}]$. However we can circumvent this by using our previous results. If we set

$$\begin{aligned} \sigma ^\mathcal R(x):=\sigma (x){{\mathrm{\mathbb {1}}}}_{\{|\sigma (x)|<\mathcal R\}}-m_\mathcal R\end{aligned}$$

we can consider the field

$$\begin{aligned} \Xi _{n,\,\mathcal R}(x):=\frac{4\pi ^2}{2d}{n^{\frac{d-4}{2}}}\sum _{z\in \mathbb {T}^d_n}\sum _{w\in {{\mathrm{\mathbb {Z}}}}_n^d}g(w,\,nz)\sigma ^\mathcal R(w){{\mathrm{\mathbb {1}}}}_{B(z,\,\frac{1}{2n})}(x),\quad x\in \mathbb {T}^d. \end{aligned}$$

Since $(2d)^{-1}\sum _{y\in {{\mathrm{\mathbb {Z}}}}_n^d}g(\cdot ,\,y)$ is a constant function on ${{\mathrm{\mathbb {Z}}}}_n^d$ it follows that

$$\begin{aligned} \left\langle \Xi _{n,\,\mathcal R},\,u \right\rangle =\left\langle \Xi _{w_n^{<\mathcal R}},\,u\right\rangle \end{aligned}$$

for all smooth functions *u* with zero average. Hence the field $\Xi _{n,\,\mathcal R}$ has the same law of $\Xi _{w_n^{<\mathcal R}}$. If we multiply and divide the former by $\sqrt{v_\mathcal R}$, we obtain

$$\begin{aligned} \Xi _{n,\,\mathcal R}=\sqrt{v_\mathcal R}\frac{4\pi ^2}{2d}{n^{\frac{d-4}{2}}}\sum _{z\in \mathbb {T}^d_n}\sum _{w\in {{\mathrm{\mathbb {Z}}}}_n^d}g(w,\,nz)\frac{\sigma ^\mathcal R(w)}{\sqrt{v_\mathcal R}}{{\mathrm{\mathbb {1}}}}_{B(z,\,\frac{1}{2n})}(x),\quad x\in \mathbb {T}^d. \end{aligned}$$

Since now the weights ${\sigma ^\mathcal R(w)}(v_\mathcal R)^{-\frac{1}{2}}$ satisfy the assumptions of Theorem 2, we know that the above field will converge to $\sqrt{v_\mathcal R}\,\Xi$ in law. Using the covariance structure of the limiting field, the fact that the field is Gaussian, and $\lim _{\mathcal R\rightarrow +\infty }\sqrt{v_\mathcal R}= 1$, a straightforward computation shows that $\sqrt{v_\mathcal R}\,\Xi$ converges in law to $\Xi$ in the topology of ${\mathcal {H}}_{-\epsilon }$. With Theorem 14 we can conclude.

## Proof of Theorem 3

*Preliminaries* We must conclude with the proof of Theorem 3 and begin by introducing some notation. We take $\zeta$, an (arbitrary) smooth radial function on ${{\mathrm{\mathbb {R}}}}^d$, such that

6.1 $$\begin{aligned} {\left\{ \begin{array}{ll} \zeta (x)=1 &{}\Vert x\Vert \ge \frac{1}{2},\\ \zeta (x)=0 &{} \Vert x\Vert \le \frac{1}{4}. \end{array}\right. } \end{aligned}$$

Let us call

$$\begin{aligned} G(x):=\zeta (x)\Vert x\Vert ^{-4}=\Vert x\Vert ^{-4}+(\zeta (x)-1)\Vert x\Vert ^{-4} \end{aligned}$$

and let ${\mathcal {G}}_d$ be its Fourier transform (in the sense of distributions)

$$\begin{aligned} {\mathcal {G}}_d(x):= \widehat{G}(x). \end{aligned}$$

Since $(\zeta (\cdot )-1)\Vert \cdot \Vert ^{-4}$ is a compactly supported distribution, its Fourier transform will be a smooth function which we call $h_d$. Using the results on $\widehat{\Vert \cdot \Vert ^{-4}}$ contained in Example 2.4.9 of Grafakos [8], we have the explicit description of ${\mathcal {G}}_d$ in (1.5). In particular ${\mathcal {G}}_d$ decays faster than the reciprocal of any polynomial function at infinity. To see this, recall that $\widehat{D^\alpha G}(x)=\left( 2\pi \iota x\right) ^{|\alpha |}{\mathcal {G}}_d(x)$, for any multi-index $\alpha$. If the order of the derivative is large enough (precisely $|\alpha |>d-4$), then $D^\alpha G(x)\in L^1({{\mathrm{\mathbb {R}}}}^d)$; in this case, $\left( 2\pi \iota x\right) ^{|\alpha |}{\mathcal {G}}_d(x)$ is bounded on ${{\mathrm{\mathbb {R}}}}^d$ and hence $\left| {\mathcal {G}}_d(x)\right| \le C \Vert x\Vert ^{-N}$ for every positive integer *N* as $\Vert x\Vert \rightarrow +\infty$. Let us denote by $f_\kappa :={\mathcal {G}}_d*\phi _\kappa$ and note that

6.2 $$\begin{aligned} \widehat{f_\kappa }(\cdot )=\widehat{{\mathcal {G}}_d}(\cdot ){\widehat{\phi _\kappa }}(\cdot )=\zeta (\cdot )\Vert \cdot \Vert ^{-4}{\widehat{\phi _\kappa }}(\cdot ). \end{aligned}$$

It follows that for some $C>0$ (depending on $\kappa$),

6.3 $$\begin{aligned} \left| \widehat{f_\kappa }(\cdot )\right| \le C(1+\Vert \cdot \Vert )^{-d-1}. \end{aligned}$$

Moreover

6.4 $$\begin{aligned} \left| f_\kappa (\cdot )\right| \le C\left( 1+\Vert \cdot \Vert \right) ^{-d-1} \end{aligned}$$

near infinity thanks to the rapid decay of ${\mathcal {G}}_d$ at infinity; furthermore ${\mathcal {G}}_d$ is integrable near zero in $d\ge 5$ by (1.5). Hence $f_\kappa$ is $C^\infty ({{\mathrm{\mathbb {R}}}}^d)$ and also in $L^1({{\mathrm{\mathbb {R}}}}^d)$. Using $f_\kappa = {\mathcal {G}}_d*\phi _\kappa$ and the definition of $\zeta$ we have that

6.5 $$\begin{aligned} (4.13)&=n^{-2d}\sum _{z,\,z'\in \mathbb {T}_n^d} u(z) u(z') \sum _{w\in {{\mathrm{\mathbb {Z}}}}_n^d}{\widehat{\phi _\kappa }}(w)\zeta (w)\frac{\exp (2\pi \iota (z-z')\cdot w)}{\Vert w\Vert ^4}\nonumber \\&=n^{-2d}\sum _{z,\,z'\in \mathbb {T}_n^d} u(z) u(z') \sum _{w\in {{\mathrm{\mathbb {Z}}}}_n^d}\widehat{f_\kappa }(w){\exp (2\pi \iota (z-z')\cdot w)}. \end{aligned}$$

Now we can rewrite this term as

6.6 $$\begin{aligned}&n^{-2d}\sum _{z,\,z'\in \mathbb {T}_n^d} u(z) u(z') \sum _{w\in {{\mathrm{\mathbb {Z}}}}^d}\widehat{f_\kappa }(w){\exp (2\pi \iota (z-z')\cdot w)}\nonumber \\&\quad -n^{-2d}\sum _{z,\,z'\in \mathbb {T}_n^d} u(z) u(z') \sum _{w\in {{\mathrm{\mathbb {Z}}}}^d:\,\Vert w\Vert _\infty >n}\widehat{f_\kappa }(w){\exp (2\pi \iota (z-z')\cdot w)}. \end{aligned}$$

First we show the second term above is negligible in the following Lemma.

### Lemma 15

$$\begin{aligned} \lim _{n\rightarrow +\infty } n^{-2d}\sum _{z,\,z'\in \mathbb {T}_n^d} u(z) u(z') \sum _{w\in {{\mathrm{\mathbb {Z}}}}^d:\,\Vert w\Vert _\infty >n}\widehat{f_\kappa }(w)\exp (2\pi \iota (z-z')\cdot w)=0. \end{aligned}$$

### Proof

Note that

$$\begin{aligned}&n^{-2d}\left| \sum _{z,\,z'\in \mathbb {T}_n^d} u(z) u(z') \sum _{w\in {{\mathrm{\mathbb {Z}}}}^d:\,\Vert w\Vert _\infty>n}\widehat{f_\kappa }(w)\exp (2\pi \iota (z-z')\cdot w)\right| \\&\quad =\left| \sum _{w\in {{\mathrm{\mathbb {Z}}}}^d:\,\Vert w\Vert _\infty>n}\widehat{f_\kappa }(w) \left( n^{-d}\sum _{z\in \mathbb {T}_n^d} u(z)\exp (2\pi \iota z\cdot w)\right) \right. \\&\left. \qquad \left( n^{-d}\sum _{z'\in \mathbb {T}_n^d} u(z')\exp (-2\pi \iota z'\cdot w)\right) \right| \\&\quad \le \Vert u\Vert _{L^\infty (\mathbb {T}^d)}^2 \sum _{w\in {{\mathrm{\mathbb {Z}}}}^d:\,\Vert w\Vert _\infty>n} \left| \widehat{f_\kappa }(w)\right| \\&\quad \le C \Vert u\Vert _{L^\infty (\mathbb {T}^d)}^2 \sum _{w\in {{\mathrm{\mathbb {Z}}}}^d:\,\Vert w\Vert _\infty >n}\frac{1}{(1+\Vert w\Vert )^{d+1}}\le C \Vert u\Vert _{L^\infty (\mathbb {T}^d)}^2 n^{-1} \end{aligned}$$

thanks to (6.3) and the Euler–MacLaurin formula [1, Theorem 1]. This shows Lemma 15. $\square$

Therefore, rather than working on (6.5), we will concentrate on the first term of (6.6).

### Proof of Theorem 3

Following the proof of Proposition 5, it is enough to prove the convergence of the first term of (6.6) to the right-hand side of (1.4). Since $f_\kappa$ and ${\widehat{f_\kappa }}$ satisfy the assumptions of the Poisson summation formula [26, Corollary 2.6, Chapter VII], we apply it to (6.5) and obtain

6.7 $$\begin{aligned}&\lim _{n\rightarrow +\infty }n^{-2d}\sum _{z,\,z'\in \mathbb {T}_n^d} u(z) u(z') \sum _{w\in {{\mathrm{\mathbb {Z}}}}^d}{\widehat{f_\kappa }}(w)\exp (2\pi \iota (z-z')\cdot w)\nonumber \\&\quad =\lim _{n\rightarrow +\infty }n^{-2d}\sum _{z,\,z'\in \mathbb {T}_n^d} u(z) u(z') \sum _{w\in {{\mathrm{\mathbb {Z}}}}^d} f_\kappa ((z-z')+w)\nonumber \\&\quad = \lim _{n\rightarrow +\infty }\sum _{w\in {{\mathrm{\mathbb {Z}}}}^d} n^{-2d}\sum _{z,\, z'\in \mathbb {T}_n^d}u(z) u(z')f_\kappa ((z-z')+w). \end{aligned}$$

We would then like to exchange sum and limit and thus we shall justify the use of the dominated convergence theorem. To this purpose we need to observe that $\Vert z-z'\Vert \le \sqrt{d}$ so that $\left| \Vert z-z'+w\Vert -\Vert w\Vert \right| \le 2\sqrt{d}.$ Therefore

6.8 $$\begin{aligned}&\sum _{w\in {{\mathrm{\mathbb {Z}}}}^d} n^{-2d}\sum _{z,\, z'\in \mathbb {T}_n^d}\left| u(z) u(z')f_\kappa ((z-z')+w)\right| \nonumber \\&\quad {\mathop {\le }\limits ^{6.4}} C n^{-2d}\Vert u\Vert _{L^\infty (\mathbb {T}^d)}^2\sum _{w\in {{\mathrm{\mathbb {Z}}}}^d:\,\Vert w\Vert _\infty >\sqrt{d}}\,\sum _{z,\, z'\in \mathbb {T}_n^d}\frac{1}{(1+\Vert z-z'+w\Vert )^{d+1}}\nonumber \\&\qquad +C n^{-2d}\Vert u\Vert _{L^\infty (\mathbb {T}^d)}^2\sum _{w\in {{\mathrm{\mathbb {Z}}}}^d:\,\Vert w\Vert _\infty \le \sqrt{d}}\,\sum _{z,\, z'\in \mathbb {T}_n^d}\frac{1}{(1+\Vert z-z'+w\Vert )^{d+1}}. \end{aligned}$$

The second term can be directly bounded by a constant independent of *n*, being a finite sum. As for the first term in (6.8) we have by the Euler–MacLaurin formula

6.9 $$\begin{aligned}&Cn^{-2d}\Vert u\Vert _{L^\infty (\mathbb {T}^d)}^2\sum _{w\in {{\mathrm{\mathbb {Z}}}}^d:\,\Vert w\Vert _\infty>\sqrt{d}}\,\sum _{z,\, z'\in \mathbb {T}_n^d}\frac{1}{(1+\Vert z-z'+w\Vert )^{d+1}}\nonumber \\&\quad \le C n^{-2d}\Vert u\Vert _{L^\infty (\mathbb {T}^d)}^2\sum _{w\in {{\mathrm{\mathbb {Z}}}}^d:\,\Vert w\Vert _\infty >\sqrt{d}}\,\sum _{z,\, z'\in \mathbb {T}_n^d}\frac{1}{(1-2\sqrt{d}+\Vert w\Vert )^{d+1}}\nonumber \\&\quad \le C\left( \int _{\sqrt{d}-1}^{+\infty }\frac{\rho ^{d-1}}{(1-2\sqrt{d}+\rho )^{d+1}}{{\mathrm{d}}}\rho +c\right) \le c \end{aligned}$$

where $C,\,c$ are independent of *n* in each occurence above. These inequalities plugged into (6.8) give the desired bound which allows us to switch summation and limit in (6.7). Going on and using also the smothness of $f_\kappa$ we compute

$$\begin{aligned}&\lim _{n\rightarrow +\infty }\sum _{w\in {{\mathrm{\mathbb {Z}}}}^d} n^{-2d}\sum _{z,\, z'\in \mathbb {T}_n^d}u(z) u(z')f_\kappa ((z-z')+w)\\&\quad =\sum _{w\in {{\mathrm{\mathbb {Z}}}}^d} \iint _{\mathbb {T}^d\times \mathbb {T}^d} u(z)u(z')f_\kappa ((z-z')+w){{\mathrm{d}}}z{{\mathrm{d}}}z'. \end{aligned}$$

The fast decay of ${\mathcal {G}}_d$ and hence of $f_\kappa$ at infinity enables us to apply the dominated convergence again to finally arrive at

$$\begin{aligned}&\lim _{\kappa \rightarrow 0}\sum _{w\in {{\mathrm{\mathbb {Z}}}}^d} \iint _{\mathbb {T}^d\times \mathbb {T}^d} u(z)u(z')f_\kappa ((z-z')+w){{\mathrm{d}}}z{{\mathrm{d}}}z'\\&\quad =\sum _{w\in {{\mathrm{\mathbb {Z}}}}^d} \iint _{\mathbb {T}^d\times \mathbb {T}^d} u(z)u(z'){\mathcal {G}}_d((z-z')+w){{\mathrm{d}}}z{{\mathrm{d}}}z'. \end{aligned}$$

Due to polynomial decay of ${\mathcal {G}}_d$ at infinity it is immediate to exchange sum and integrals to derive (1.4). $\square$
